# Hypergraph learning with multi-dimensional metabolite feature extractions and static–dynamic attention mechanisms to fill missing reactions in metabolic networks

**DOI:** 10.1093/bib/bbag314

**Published:** 2026-06-17

**Authors:** Kai Wang, Jiajun Qu, Fei Liu, Xiaoli Luan, Jingwen Zhou

**Affiliations:** Key Laboratory of Advanced Process Control for Light Industry (Ministry of Education), School of Automation and Intelligent Science (School of Internet of Things), Jiangnan University, 1800 Lihu Road, Wuxi, Jiangsu 214122, China; Key Laboratory of Advanced Process Control for Light Industry (Ministry of Education), School of Automation and Intelligent Science (School of Internet of Things), Jiangnan University, 1800 Lihu Road, Wuxi, Jiangsu 214122, China; Key Laboratory of Advanced Process Control for Light Industry (Ministry of Education), School of Automation and Intelligent Science (School of Internet of Things), Jiangnan University, 1800 Lihu Road, Wuxi, Jiangsu 214122, China; Key Laboratory of Advanced Process Control for Light Industry (Ministry of Education), School of Automation and Intelligent Science (School of Internet of Things), Jiangnan University, 1800 Lihu Road, Wuxi, Jiangsu 214122, China; Science Center for Future Foods, Jiangnan University, 1800 Lihu Road, Wuxi, Jiangsu 214122, China; Key Laboratory of Industrial Biotechnology, Ministry of Education and School of Biotechnology, Jiangnan University, 1800 Lihu Road, Wuxi, Jiangsu 214122, China; Engineering Research Center of Ministry of Education on Food Synthetic Biotechnology, Jiangnan University, 1800 Lihu Road, Wuxi, Jiangsu 214122, China; Jiangsu Province Engineering Research Center of Food Synthetic Biotechnology, Jiangnan University, 1800 Lihu Road, Wuxi, Jiangsu 214122, China

**Keywords:** genome-scale metabolic model, gap-filling, hypergraph learning, pretrained language model, static–dynamic attention mechanism

## Abstract

Genome-scale metabolic models (GEMs) can effectively facilitate many fields in synthetic biology, biomanufacturing, and biomedicine. Reconstructing high-quality GEMs is crucial for accurate phenotype predictions of organisms. However, draft GEMs generated by automated reconstruction tools contain many knowledge gaps, especially missing reactions. The existing machine learning-based gap-filling approaches need to be further developed. In this article, we propose a novel HyperGraph Learning approach with Multi-dimensional metabolite feature extractions and static–dynamic Attention mechanisms (HGLMA) for predicting and teasing out missing reactions in GEM gap-fillings. HGLMA simultaneously uses two pretrained language models to proceed multi-dimensional metabolite feature extractions, which are further fused and regarded as node embeddings for graph learning. The directed and high-order associations between metabolites in reactions of GEMs are deeply mined by successively employing a directional graph network and a hypergraph neural network. Before outputting the predicted confidence score for candidate reactions, the static–dynamic multi-head attention mechanism is utilized to automatically learn attention weights and to identify key metabolites within any candidate reaction. The five-fold cross-validation results on 108 BiGG GEMs show that HGLMA significantly outperforms other state-of-the-art machine learning-based approaches both in prediction performances and in the ability of discovering missing reactions from metabolic reaction pools. The ablation study shows the contributions of multi-dimensional feature extractions and static–dynamic attention mechanisms. In addition, the phenotype prediction results of 24 bacterial organisms demonstrate the effectiveness and superiority of gap-fillings by HGLMA.

## Introduction

Genome-scale metabolic models (GEMs) are powerful tools of understanding cellular metabolisms and simulating physiological behaviors in bioinformatics and synthetic biology [1, 2]. The GEM of a living organism associates metabolites with reactions by the stoichiometric matrix, and associates reactions with genes and corresponding enzymes by the reaction–gene matrix, so as to systematically describe the gene–protein–reaction (GPR) rules [3–5]. GEMs reveal the underlying mechanisms from genotype to phenotype, and can be used to predict cellular metabolic behaviors in different environments and conditions through flux balance analysis [6–8]. Nowadays, GEMs have been effectively applied to many fields of biomanufacturing and biomedicine, such as the cellular phenotype analysis [9], rational design of chassis strains [10], metabolic engineering [11, 12], therapeutic target identification [13], drug discovery [14], etc. Therefore, it is crucial to reconstruct high-quality GEMs. Recently, numerous automated reconstruction tools, such as ModelSEED [15], CarveMe [16], gapseq [17], Merlin [18], etc., have been developed to accelerate the GEM reconstruction process, by making use of genome annotations, underlying metabolic reactions in reference databases, etc. However, the draft GEMs generated by these reconstruction tools inevitably contain many knowledge gaps, due to inaccurate genomic and functional annotations, unknown reactions and pathways, etc. Therefore, it is necessary to further conduct gap-fillings for draft GEMs, in order to guarantee the physiological prediction accuracy of metabolic network models [19, 20].

To fill the knowledge gaps in draft GEMs, various automated gap-filling tools have been proposed. The traditional optimization-based gap-filling approaches, including GrowMatch [21], MIRAGE [22], etc., mainly aim to identify inconsistencies between model predictions and experimental data and to further resolve the inconsistencies by integrating additional reactions. The dependencies on phenotypic data limit the practical utility of these approaches, especially for non-model organisms. Moreover, there are some classical topology-based gap-filling tools without the need of phenotypic data, such as FASTGAPFILL [23], Meneco [24], etc. However, it is difficult for these methods to effectively achieve accurate gap-filling results, especially for large-scale GEMs.

During these years, machine learning algorithms have been employed to identify and fill missing reactions in draft GEMs. Many of these state-of-the-art machine learning-based approaches formulate the gap-filling task as a hyperedge (or called hyperlink) prediction problem for the hypergraph of a GEM [25, 26]. For instance, C3MM [27] is the hyperedge prediction algorithm based on a proposed clique-closure hypothesis. NHP [28] utilizes graph convolutional networks (GCNs) for predicting candidate reactions that can be missing from metabolic networks, and its variant NHP-D can achieve hyperedge predictions in directed hypergraphs. CHESHIRE [29] leverages the Chebyshev spectral graph convolutional network (CSGCN) for feature refinements, and has been used for gap-fillings according to prediction scores of candidate reactions. The phenotype prediction results show the effectiveness of gap-fillings by CHESHIRE. HGNNP [30] is a recent hypergraph neural network used for hyperedge predictions, which is effective in mining high-order correlations. DSHCNet [31] employs dual-scale fused hypergraph convolution methods for predicting missing reactions, which can effectively distinguishes substrates and products. It should be noted that the node feature embeddings of the above approaches are obtained purely from the GEM topology without using the information of metabolites. CLOSEgaps [32] captures topological features through the hypergraph mapping and the similarity matrix of metabolites, and uses hypothetical reactions to identify missing reactions within the network. Multi-HGNN [33] extracts metabolite biochemical features according to molecular graphs and uses these extracted feature embeddings for further hypergraph learning. MuSHIN [34] is the most state-of-the-art hypergraph-learning-based method of predicting missing metabolic reactions for draft GEMs. This method utilizes the SMILES of reactions and metabolites combined with hypergraph learning to predict and fill missing reactions in GEMs. However, it is still necessary to further exploit the features of metabolites in GEMs and candidate reactions, such that the performances of machine learning-based gap-filling approaches can be further improved.

To address these issues, we propose a novel hypergraph learning approach with multi-dimensional metabolite feature extractions and static–dynamic multi-head attention mechanisms, called HGLMA, for teasing out missing reactions in GEM fillings. The confident scores of candidate metabolic reactions as missing gaps of a given GEM can be predicted by HGLMA according to the input GEM topology and metabolite information. The prediction results can be further used for gap-fillings together with universal reaction pools. In the metabolite feature extraction module, we simultaneously utilize two molecular pretrained models ChemBERTa [35] and GraphMVP [36] to, respectively, extract the multi-dimensional features of the input SMILES sequences and molecular graphs of metabolites. These feature representations are further properly fused and used as initial node embeddings. This method can fully exploit metabolite features by extracting 1D and 2D molecular features, and can implicitly extract 3D features through GraphMVP. The hypergraph learning module successively employs a directional graph network (DGN) [37] and a hypergraph neural network (HGNNP) [30] to extract the metabolite and topology features of GEMs, where both directed and high-order metabolic associations within and between reactions can be fully mined. In the attention mechanism module, we leverage a static–dynamic multi-head mechanism strategy to automatically assign attention weights and to identify key metabolites within any candidate reaction, and finally output the predicted confidence score of the reaction. We evaluated reaction prediction performances of our proposed HGLMA and five other state-of-the-art baseline approaches, by conducting five-fold cross-validations on 108 BiGG GEMs. The results show that HGLMA significantly outperforms other baseline methods in terms of AUPRC, recall, F1 score, and accuracy metrics. Furthermore, the reaction recovery experiments were carried out for the 108 GEMs. The experiment results demonstrate that HGLMA can obviously improve the ability of discovering missing reactions from various scales of real metabolic reactions. The ablation experiments show the contributions of multi-dimensional metabolite feature extractions and static–dynamic multi-head attention mechanisms in HGLMA for missing reaction predictions. Finally, we conducted gap-fillings for draft GEMs of 24 bacteria, and used COBRApy to conduct metabolic phenotype predictions of fermentation products. By regarding the experimental fermentation phenotype data (available in [17]) as the baseline, the prediction results demonstrate the effectiveness of our gap-filling approach (HGLMA) for reconstructing more accurate GEMs.

## Materials and methods

### Datasets

In this study, the datasets were conducted so as to train and to test our proposed approach of predicting and filling missing reactions in GEMs. According to previous related studies in [29, 31, 33], we selected 108 benchmark GEMs from the BiGG database [38]. Furthermore, for any one of GEMs, each reaction was treated as a positive sample (assigned label 1). A metabolite node from a positive sample reaction was randomly selected and replaced with one of 10 393 metabolites in the BiGG database, generating the same number of negative reaction samples (assigned label 0). Moreover, following previous studies in [29], we chose an universal BiGG reaction pool consisting of 16 337 metabolic reactions to tease out missing reactions in GEMs, after the training phase of our proposed approach. The SMILES sequences [39] of the forementioned metabolites were retrieved from the MetaNetX website [40]. RDKit was used to convert the SMILES sequences of metabolites into corresponding 2D molecular graph representations (see https://www.rdkit.org).

### Model architecture

#### Overall framework

**Figure 1.**
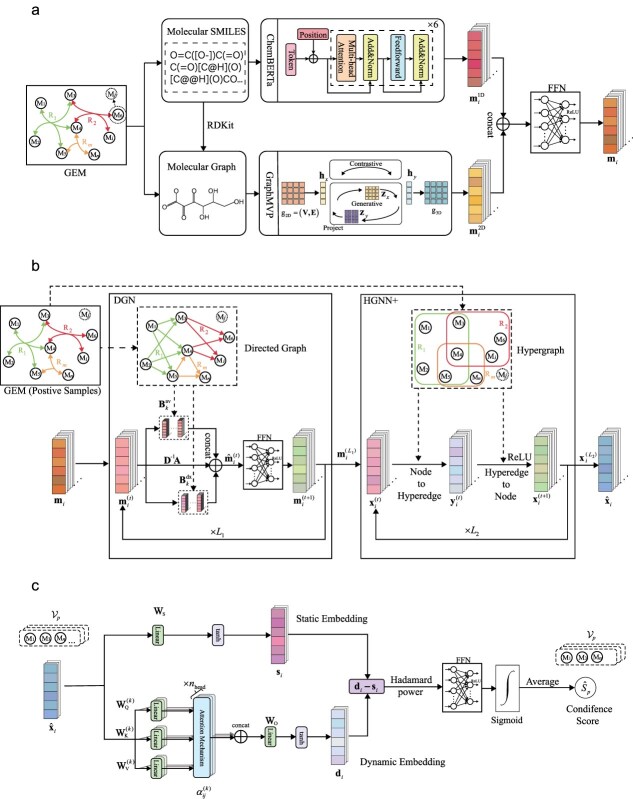
Overall architecture of the proposed HGLMA. (a) Metabolite feature extraction module. (b) Hypergraph learning module. (c) Attention mechanism module.

The overall architecture of the proposed reaction prediction approach, HGLMA, is shown in Fig. 1. This method consists of three main modules, which are the metabolite feature extraction module, hypergraph learning module, and attention mechanism module.

- Metabolite feature extraction module: This module is to obtain initial feature embeddings of metabolites corresponding to the GEM, by simultaneously extracting multi-dimensional features of metabolites through pretrained language models and fusing these features.
- Hypergraph learning module: This module further processes the initial feature embeddings of metabolites, to obtain refined metabolite feature embeddings with directed and high-order interaction information by using hypergraph learning approaches with the directed graph and hypergraph of the GEM.
- Attention mechanism module: This module learns the attention weights of metabolites in any prediction reaction by employing a static–dynamic multi-head attention mechanism to further identify key metabolite nodes, and finally outputs the confidence score for any prediction reaction.

#### Metabolite feature extraction module

Suppose that there are $n$ distinct metabolites in all the positive and negative reaction samples and candidate reactions, which are regarded as $n$ nodes of the corresponding GEM. This module extracts the multi-dimensional features of these metabolites by simultaneously feeding their SMILES sequences and molecular graph representations into two corresponding molecular pretrained language models and fuses the multi-dimensional feature representations.

According to SMILES sequences of $n$ metabolites, we utilize ChemBERTa [35], a molecular pretrained model, to extract 1D molecular features of metabolites, which yields the corresponding feature representation vectors $\mathbf{m}_{i}^{\mathrm{1D}} \in \mathbb{R}^{N_{1}}$ for $i = 1, 2, \ldots , n$. Specifically, ChemBERTa is based on the BERT architecture with byte-level BPE tokenization approach [41], where there are totally six encoder layers and each layer comprises 12 attention heads. It utilizes the RoBERTa pretraining method [42] with a random masking strategy and has been pretrained from compounds in ZINC database [43].

Simultaneously, we feed molecular graph representations of metabolites, which are obtained from SMILES sequences by RDKit, into a pretrained GraphMVP [36]. This can extract 2D molecular features and implicitly capture 3D molecular features. We obtain the corresponding feature representation vectors $\mathbf{m}_{i}^{\mathrm{2D}} \in \mathbb{R}^{N_{2}}$ for $i = 1, 2, \ldots , n$. Any molecular graph can be represented as a graph object structure, described by the atomic attribute matrix and bond attribute matrix (see https://pytorch-geometric). Specifically, GraphMVP employs a graph multi-view pretraining framework for molecular encoding, and performs self-supervised learning by leveraging the correspondence and consistency between 2D topological structures and 3D geometric views. It has been pretrained on 50k randomly selected qualified molecules with both 2D and 3D structures from the GEOM database [44], which can form implicit 3D feature representations of metabolite molecules through their 2D graph representations.

By concatenating $\mathbf{m}_{i}^{\mathrm{1D}}$ and $\mathbf{m}_{i}^{\mathrm{2D}}$ and passing through a single-layer feedforward neural network (FFN) with sigmoid activation functions, we obtain the initial metabolite feature embeddings $\mathbf{m}_{i} \in \mathbb{R}^{N_{3}}$ as


(1) \begin{eqnarray*}& \mathbf{m}_{i} = \mathrm{ReLU} \big(\mathbf{W}_{1} \mathrm{concat}(\mathbf{m}_{i}^{\mathrm{1D}}, \mathbf{m}_{i}^{\mathrm{2D}}) + \mathbf{b}_{1} \big),\end{eqnarray*}

where $i=1,2,...,n$, $\mathbf{W}_{1}$ is a learnable weight matrix, and $\mathbf{b}_{1}$ is a learnable bias vector.

#### Hypergraph learning module

This module is to further proceed the GEM feature extractions through hypergraph learning approaches. This process is based on the topological information of the input GEM consisting of $n$ metabolites (discussed as above) and $m$ reactions (only positive samples).

The structure of the input GEM can be represented by a directed graph $\mathcal{G}=(\mathcal{V}, \mathcal{E})$, where $\mathcal{V}=\{ M_{1}, M_{2}, \ldots , M_{n} \}$ is the set of $n$ metabolite nodes, and $\mathcal{E}=\{ E_{1}, E_{2}, \ldots , E_{l} \}$ is the set of directed edges indicating substrate–product relationships in positive reaction samples. According to Multi-HGNN [33], reversible reactions are treated as unidirectional reactions and compartmentalized metabolites are treated as distinct nodes. Therefore, the directed graph $\mathcal{G}$ can describe metabolic associations within and between reactions in a GEM. From the adjacency matrix $\mathbf{A}\in \mathbb{R}^{n \times n}$ of $\mathcal{G}$, the degree diagonal matrix $\mathbf{D}\in \mathbb{R}^{n \times n}$ and the Laplacian matrix $\mathbf{L}=\mathbf{D}-\mathbf{A}$ can be calculated. Then, according to the initial feature embeddings $\mathbf{m}_{i}$ for $i=1, 2, \ldots , n$ and the directed graph $\mathcal{G}$ of the GEM, we employ an $L_{1}$-layer DGN [37] to further obtain the feature representation vectors $\mathbf{x}_{i} \in \mathbb{R}^{N_{4}}$ for $i=1, 2, \ldots , n$, where each layer mainly conducts feature aggregations followed by a single-layer FFNs with ReLU activation functions. The above operations can be presented as


(2) \begin{eqnarray*} & \hat{\mathbf{m}}_{i}^{(t)} = \mathrm{concat}\left(\left[\mathbf{D}^{-1}\mathbf{AM}^{(t)} \right]_{i,:}^{\mathrm{T}}, \left[|\mathbf{B}_{1}^{\mathrm{dx}}\mathbf{M}^{(t)}|\right]_{i,:}^{\mathrm{T}}, \right. \nonumber \\ & \left. \left[\mathbf{B}_{1}^{\mathrm{av}}\mathbf{M}^{(t)}\right]_{i,:}^{\mathrm{T}}, \ldots, \left[|\mathbf{B}_{K}^{\mathrm{dx}}\mathbf{M}^{(t)}|\right]_{i,:}^{\mathrm{T}}, \left[\mathbf{B}_{K}^{\mathrm{av}}\mathbf{M}^{(t)}\right]_{i,:}^{\mathrm{T}}\right)\kern-2pt, \end{eqnarray*}

(3) \begin{eqnarray*} & \mathbf{m}_{i}^{(t+1)} = \mathrm{ReLU} \left(\mathbf{W}_{2}^{(t)} \hat{\mathbf{m}}_{i}^{(t)} + \mathbf{b}_{2}^{(t)}\right)\kern-2pt, \end{eqnarray*}

and


(4) \begin{eqnarray*}& \mathbf{x}_{i} = \mathbf{m}_{i}^{(L_{1})},\end{eqnarray*}

where $i = 1, 2, \dots , n$, $t = 0, 1, \ldots , L_{1} - 1$, $\mathbf{M}^{(t)} = \left [ \mathbf{m}_{1}^{(t)} \ \ \mathbf{m}_{2}^{(t)} \ \ \ldots \ \ \mathbf{m}_{n}^{(t)} \right ]^{\mathrm{T}}$, $\mathbf{m}_{i}^{(0)} = \mathbf{m}_{i}$, and $\mathbf{W}_{2}^{(t)}$ and $\mathbf{b}_{2}^{(t)}$ are, respectively, learnable weight matrices and bias vectors. In the above equations, $[\cdot ]_{i,:}$ denotes the $i$th row of matrix $\cdot$, $|\cdot |$ denotes the absolute values of elements in a matrix $\cdot$. Moreover, $\mathbf{B}_{k}^{\mathrm{av}} \in \mathbb{R}^{n \times n}$ and $\mathbf{B}_{k}^{\mathrm{dx}} \in \mathbb{R}^{n \times n}$ for $k = 1, 2, \dots , K$, respectively, denote the directed smooth matrices and the directed derivative matrices of the directed graph $\mathcal{G}$, and can be determined by the Laplacian matrix $\mathbf{L}$, where the details of the calculation process can be referred to Supplementary Note S1.

Moreover, the topological structure of the input GEM can be also represented by a hypergraph $\mathcal{H}=(\mathcal{V},\mathcal{R})$, where $\mathcal{V}=\{ M_{1}, M_{2}, \ldots , M_{n} \}$ is the set of $n$ metabolite nodes, and $\mathcal{R}=\{ R_{1}, R_{2}, \ldots , R_{m} \}$ is the set of hyperedges indicating reactions in all positive reaction samples. According to Multi-HGNN [33], compartmentalized metabolites are treated as distinct nodes. Therefore, the hypergraph $\mathcal{H}$ can describe the high-order interaction relationship information between reactions and metabolites in a GEM. From the hypergraph structure $\mathcal{H}=(\mathcal{V},\mathcal{R})$ and incidence matrix $\mathbf{H} \in \mathbb{R}^{n \times m}$, we can directly calculate the diagonal matrix $\mathbf{D}_{1} \in \mathbb{R}^{m \times m}$ describing hyperedge degrees and the diagonal matrix $\mathbf{D}_{2} \in \mathbb{R}^{n \times n}$ describing node degrees. Then, we feed the feature representations obtained from DGN and the hypergraph $\mathcal{H}$ of the GEM into an $L_{2}$-layer hypergraph neural network called $\mathrm{HGNNP}$ [30] to conduct hypergraph learning. In each layer, hyperedge feature representations $\mathbf{Y}^{(t)} \in \mathbb{R}^{N_{4}}$ are obtained by aggregating neighbor node features, and node features $\mathbf{X}^{(t+1)} \in \mathbb{R}^{N_{5}}$ are then updated by aggregating neighbor hyperedge features. The above process can be summarized as


(5) \begin{eqnarray*}& \mathbf{Y}^{(t)} = \mathbf{D}_{1}^{-1}\mathbf{H}^{\mathrm{T}}\mathbf{X}^{(t)},\end{eqnarray*}

and


(6) \begin{eqnarray*}& \mathbf{X}^{(t+1)} = \mathrm{ReLU}\left(\mathbf{D}_{2}^{-1}\mathbf{H}\mathbf{Y}^{(t)}\mathbf{W}_{3}^{(t)\mathrm{T}}\right),\end{eqnarray*}

where $t = 0, 1, \ldots , L_{2}-1$, $\mathbf{X}^{(0)}=\mathbf{X} = \left [ \mathbf{x}_{1} \quad \mathbf{x}_{2} \quad \dots \quad \mathbf{x}_{n} \right ]^{\mathrm{T}}$, and $\mathbf{W}_{3}^{(t)}$ are learnable weight matrices. The refined feature embeddings $\hat{\mathbf{x}}_{i} \in \mathbb{R}^{N_{5}}$ for $i = 1, 2, \ldots , n$ are obtained by transposing all the row vectors of $\mathbf{X}^{(L_{2})}$:


(7) \begin{eqnarray*}& \hat{\mathbf{X}} \triangleq \left[ \hat{\mathbf{x}}_{1} \quad \hat{\mathbf{x}}_{2} \quad \dots \quad \hat{\mathbf{x}}_{n} \right]^{\mathrm{T}} = \mathbf{X}^{(L_{2})}.\end{eqnarray*}

#### Attention mechanism module

This module uses a static–dynamic multi-head attention mechanism [45] to further process the refined feature embeddings of the metabolites in any candidate reaction, such that the attention weights of metabolites can be automatically determined, and finally outputs the confidence score of predicting the candidate reaction.

For any candidate reaction whose metabolites form a node set $\mathcal{V}_{p} \subseteq \mathcal{V}$, letting the feature embeddings $\hat{\mathbf{x}}_{i}$ for $M_{i} \in \mathcal{V}_{p}$ pass through a positional FFN, we obtain the static embeddings $\mathbf{s}_{i} \in \mathbb{R}^{N_{5}}$ as


(8) \begin{eqnarray*}& \mathbf{s}_{i} = \tanh(\mathbf{W}_{\mathrm{S}} \hat{\mathbf{x}}_{i}),\end{eqnarray*}

where $\mathbf{W}_{\mathrm{S}}$ is a learnable static embedding weight matrix. Simultaneously, we employ a multi-head attention layer with $n_{\mathrm{head}}$ attention heads to obtain the dynamic embeddings $\mathbf{d}_{i} \in \mathbb{R}^{N_{5}}$ of $M_{i} \in \mathcal{V}_{p}$ as


(9) \begin{eqnarray*} \mathbf{d}_{i} = &\tanh\left(\mathbf{W}_{\mathrm{O}} \mathrm{concat}\left( \sum_{\substack{M_{j} \in \mathcal{V}_{p} \\ j \neq i}} \alpha^{(1)}_{ij} \mathbf{W}^{(1)}_{\mathrm{V}}\hat{\mathbf{x}}_{j}, \right.\right.\nonumber\\ & \left.\left. \ldots, \sum_{\substack{M_{j} \in \mathcal{V}_{p} \\ j \neq i}} \alpha^{(n_{\mathrm{head}})}_{ij} \mathbf{W}^{(n_{\mathrm{head}})}_{\mathrm{V}}\hat{\mathbf{x}}_{j} \right)\right), \end{eqnarray*}

where


(10) \begin{eqnarray*}& \alpha_{ij}^{(k)} = \frac{\exp \left( \left( \mathbf{W}_{\mathrm{Q}}^{(k)} \hat{\mathbf{x}}_{i} \right)^{\mathrm{T}} \left( \mathbf{W}_{\mathrm{K}}^{(k)} \hat{\mathbf{x}}_{j} \right) \right)} {\sum\limits_{M_{l} \in \mathcal{V}_{p}} \exp \left( \left( \mathbf{W}_{\mathrm{Q}}^{(k)} \hat{\mathbf{x}}_{i} \right)^{\mathrm{T}} \left( \mathbf{W}_{\mathrm{K}}^{(k)} \hat{\mathbf{x}}_{l} \right) \right)},\end{eqnarray*}

$k = 1, 2, \ldots , n_{\mathrm{head}}$ , $\mathbf{W}_{\mathrm{O}}$ is a learnable output matrix, and $\mathbf{W}_{\mathrm{V}}^{(k)}, \mathbf{W}^{(k)}_{\mathrm{Q}}$, and $\mathbf{W}^{(k)}_{\mathrm{K}}$ are, respectively, learnable value, query and key matrices. Finally, the Hadamard square of the difference between the static and dynamic embedding vectors is calculated and fed into a single-layer FFN with sigmoid activation functions. This can yield the probability scores corresponding to metabolite nodes belong to $\mathcal{V}_{p}$. The above process is presented as


(11) \begin{eqnarray*}& \hat{P}_{i} = \mathrm{sigmoid}\!\left( \mathbf{W}_{4} (\mathbf{d}_{i} - \mathbf{s}_{i})^{\circ 2} + \mathbf{b}_{4} \right)\kern-2pt,\end{eqnarray*}

where $M_{i} \in \mathcal{V}_{p}$, $\mathbf{W}_{4}$ is a learnable weight matrix, and $\mathbf{b}_{4}$ is a learnable bias vector.

Finally, we obtain the confidence score $\hat{S}_{p} \in [0,1]$ for the candidate reaction formed by metabolites in $\mathcal{V}_{p}$, which is derived by taking the average value of $\hat{P}_{i}$ across all metabolites in $\mathcal{V}_{p}$:


(12) \begin{eqnarray*}& \hat{S}_{p} = \frac{1}{|\mathcal{V}_{p}|} \sum_{M_{i} \in \mathcal{V}_{p}} \hat{P}_{i},\end{eqnarray*}

where $|\mathcal{V}_{p}|$ denotes the number of nodes in $\mathcal{V}_{p}$.

### Model training

For the training of the proposed HGLMA, we employ the cross-entropy loss function to calculate the loss between the true labels and confidence values of reaction samples in the $k$th batch $\mathcal{I}_{k}$ for $k = 1, 2, \cdots , n_{\mathrm{batch}}$:


(13) \begin{eqnarray*} \mathcal{L} = - \frac{1}{|\mathcal{I}_{k}|} \sum_{p \in \mathcal{I}_{k}} \left( S_{p} \log \hat{S}_{p} + (1 - S_{p}) \log (1 - \hat{S}_{p}) \right),\end{eqnarray*}

where $|\mathcal{I}_{k}|$ denotes the batch size, $S_{p}$ is the label of the $p$th reaction and $\hat{S}_{p}$ is the confidence score of the $p$th reaction that is predicted by HGLMA (see Equations 1–12). The proposed model can be implemented and trained by using Python 3.8 and PyTorch 2.1.0 with CUDA 12.1 and an NVIDIA 4090 graphics card. HGLMA has 4.208 million parameters with the maximal GPU memory usage of 178.80 MB. In the training process, we individually conducted the trainings for each GEM and the Adam optimization algorithm [46] was employed to adjust the learnable parameters by minimizing the loss function as in Equation (13) with an initial learning rate of $0.0005$. The details of learnable parameter matrices/vectors and the details of hyperparameter values are, respectively, presented in Supplementary Tables S1 and S2. Moreover, Supplementary Fig. S3 presents the reaction prediction performance results of our random negative sampling approach compared with those negative sampling methods by randomly selecting real negative samples from the BiGG universal reaction pool for the training or testing samples on iAF1260b and Recon3D GEMs. The comparison experiment results demonstrate the rationality of the negative sampling approach utilized in this paper.

### Gap-filling by the model

Consider a draft GEM of an organism, which can be reconstructed from genome sequences by using automated GEM reconstruction tools. We can make use of the corresponding positive and negative training samples and graph descriptions of the draft GEM to train the proposed HGLMA by following the previous discussions. We can individually train our proposed HGLMA by using each draft GEM (all the reactions and corresponding negative sampling reactions) and use trained HGLMA model to predict the confidence scores of all the metabolic reactions in a candidate reaction pool for each draft GEM, and exclude those reactions that already exist in the draft GEM. Then, we choose the candidate reactions whose confidence scores exceed a specific threshold, such as 0.9995.

Furthermore, according to Chen *et al*. [29], we obtain reaction similarity scores by calculating the Pearson correlation coefficient between each candidate reaction selected as described above and the existing reactions in the draft GEM. The reactions with lower similarity are more likely to be complementary to the current model. Finally, the chosen candidate reactions are sorted according to reaction similarity scores in ascending order, and the top $K$ reactions are selected for gap-fillings.

The above process can be illustrated in Supplementary Fig. S1.

## Results

### Baseline models

For performance comparisons of our proposed HGLMA model with other related state-of-the-art approaches, we selected C3MM [27], NHP [28], CHESHIRE [29], HGNNP [30], and Multi-HGNN [33] as baseline models. To evaluate performances of reaction predictions and reaction recovery of GEMs, we compared our proposed HGLMA with all these five baseline methods. In addition, HGLMA was compared with CHESHIRE [29], CLOSEgaps [32], and MuSHIN [34] to assess the effectiveness of gap-fillings through metabolic phenotype predictions.

### Evaluation of reaction prediction performance

In order to evaluate the ability of predicting candidate metabolic reactions by our proposed HGLMA model, we conducted the training and testing (five-fold cross-validation) for each GEM separately to obtain its corresponding performance metrics. In each of five rounds, we randomly divided any of 108 datasets into training and testing subsets at 4:1 ratio with the same number of positive and negative reaction samples. The performance metric values for each dataset were determined by taking the mean values of those in five rounds. According to previous related investigations in [29, 33], we employed four metrics to measure prediction performances, which are the area under the precision–recall curve (AUPRC), recall, F1 score, and accuracy.

The hyperparameter analysis of HGLMA on the iBWG-1329 dataset, one of the 108 BiGG GEMs, is illustrated in Supplementary Fig. S2. The results show the performance variations across a set of various values of hyperparameters. The determinations of hyperparameter values (see Supplementary Table S2) can be referred to the analysis.

**Figure 2.**
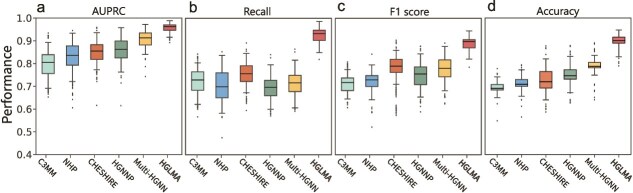
Boxplots of prediction performances of HGLMA and five baseline models on 108 BiGG GEMs, in terms of (a) AUPRC, (b) Recall, (c) F1 score, and (d) accuracy.

The distributions and average values of prediction performances of HGLMA and five baseline models on 108 BiGG GEMs are presented in Fig. 2 and Table 1, respectively. For the 108 GEMs, the details of performance metric values of HGLMA can be referred to Supplementary Table S3. Table 1 shows that the proposed HGLMA achieves the best prediction performances, in terms of average values of AUPRC, recall, F1 score, and accuracy, on 96.3% (104/108), 100% (108/108), 100% (108/108), and 90.7% (98/108) of datasets, with the relative improvements of 5.1%, 22.4%, 15.9%, and 12.6%, respectively. Moreover, the boxplots of performance distributions on 108 BiGG datasets in Fig. 2 graphically depicts that the proposed HGLMA significantly outperforms all the baseline methods in terms of these four metrics. To be specific, the median values of AUPRC, recall, F1 score, and accuracy of HGLMA are 0.963, 0.935, 0.908, and 0.905, respectively, which are 5.8%, 24%, 17.3%, and 13.7% relatively higher than the second best model (Multi-HGNN or CHESHIRE). The upper quartile (respectively lower quartile) values of AUPRC, recall, F1 score, and accuracy of HGLMA are 0.968, 0.947, 0.916, and 0.913 (respectively 0.953, 0.907, 0.882, and 0.882), respectively, which are 6.4%, 21.9%, 15.5%, and 11.5% (respectively 7.2%, 24.4%, 16.7%, and 13.1%) relatively higher than the second best model (Multi-HGNN or CHESHIRE).

**Table 1:** Average values of prediction performances of HGLMA and five baseline models on 108 BiGG GEMs

Models	AUPRC	Recall	F1 score	Accuracy
C3MM [27]	0.803	0.683	0.702	0.698
NHP [28]	0.828	0.698	0.721	0.717
CHESHIRE [29]	0.861	0.755	0.774	0.735
HGNNP [30]	0.862	0.695	0.743	0.756
Multi-HGNN [33]	0.910	0.715	0.763	0.795
**HGLMA**	**0.956 (104/108)**	**0.924 (108/108)**	**0.897 (108/108)**	**0.895 (98/108)**

In summary, the results demonstrate that our proposed HGLMA has the best prediction capacity, due to the combination of hypergraph learning with multi-dimensional metabolite feature extractions and static–dynamic multi-head attention mechanisms. In addition, the superior performances of Multi-HGNN to other four baseline models in two of four metrics indicate the effectiveness of employing metabolite feature representations as node embeddings.

**Figure 3.**
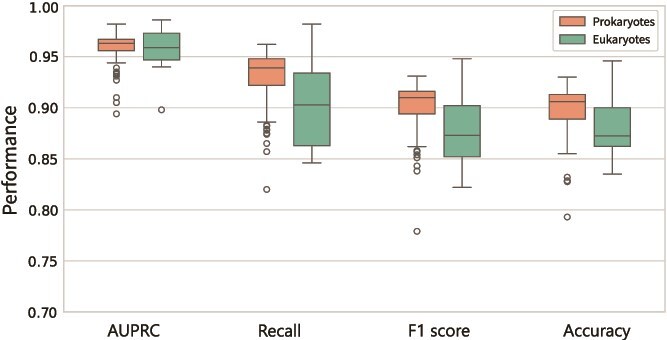
Boxplots of reaction prediction performances of HGLMA on GEMs of prokaryotes and eukaryotes.

In addition, we discussed the reaction prediction performances on prokaryotic and eukaryotic GEMs of the 108 BiGG datasets (see Supplementary Table S4), in order to verify the influence of specifies on the prediction capacity of HGLMA. The distributions of prediction performances for these two species are shown in Fig. 3. The results illustrate that HGLMA exhibits better performances in missing reaction predictions for prokaryotes than for eukaryotes, which may be attributed to more complex metabolic network structures and compartmentalizations of metabolites in eukaryotes. In spite of this, we can note that HGLMA can still provide high prediction performances for most of enkaryotic GEMs.

### Evaluation of reaction recovery

To evaluate the ability of distinguishing missing reactions from other real reactions in gap-fillings, we further conducted reaction recovery experiments on the 108 BiGG datasets. In the experiments, we used the mean values of performances in five rounds for evaluations, and dataset divisions into training and testing subsets were the same as those of five-fold cross-validations in reaction predictions. In the testing subsets of 108 GEM datasets, we removed all the negative testing samples obtained through negative samplings, randomly selected real metabolic reactions from the candidate reaction pool (totally 16 337 metabolic reactions) in BiGG datasets, and combined them with a certain number of positive testing samples as candidate reactions. Instead of applying a fixed threshold, we selected the Top 25, 50, 100, and $N$ reactions with the highest confidence scores from all candidate reactions, where $N$ denotes the number of positive testing samples in any dataset. According to Chen *et al*. [29], we used the reaction recovery rate to measure performances, which is the proportion of positive testing samples in the selected top reactions.

**Figure 4.**
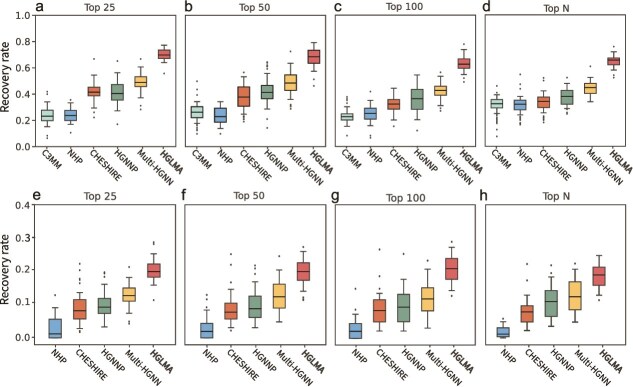
Boxplots of reaction recovery performances of HGLMA and five baseline models on 108 BiGG GEMs, by selecting Top 25, 50, 100, and $N$ reactions, respectively. (a–d) The experiment results of using candidate reactions that include positive testing samples and the same number of BiGG reactions. (e–h) The experiment results of using candidate reactions that include positive testing samples and all the 16 337 metabolic reactions.

The reaction recovery experiments were first conducted for the case when the candidate reactions include positive testing samples and the same number of other reactions randomly selected from the 16 337 metabolic reactions. For HGLMA and five baseline models, the distributions and average values of recovery rates on 108 BiGG GEMs are presented in Fig. 4a–d and the top part of Table 2, respectively. The detailed experiment results can be referred to Supplementary Table S5. It can be seen from Table 2 that HGLMA achieves the best recovery rate, on 100% (108/108), 100% (108/108), 94.4% (102/108), and 90.7% (98/108) of 108 datasets, and improves 38.9%, 38.9%, 49.9%, and 47.9% compared with the second best model (Multi-HGNN), for the cases of selecting Top 25, 50, 100, and $N$ reactions, respectively. Moreover, Fig. 3 graphically shows that HGLMA is significantly superior to five baseline methods on 108 BiGG GEMs. For the cases of Top 25, 50, 100, and $N$, respectively, the median recovery rate values of HGLMA are 0.697, 0.675, 0.636, and 0.644, respectively, which are 38.8%, 38.3%, 48.9%, 48.4% higher than the second best model (Multi-HGNN). The upper quartile (respectively. lower quartile) values of Top 25, Top 50, Top 100, and Top $N$ of HGLMA are 0.723, 0.694, 0.645, and 0.659 (respectively 0.674, 0.648, 0.612, and 0.617), respectively, which are 38.2%, 34.5%, 42.1%, and 45.1% (respectively 41.9%, 40.6%, 51.1%, and 50.1%) higher than the second best model (Multi-HGNN).

**Table 2:** Average values of recovery rates of HGLMA and five baseline models on 108 BiGG GEMs

Candidate reactions	Models	Top 25	Top 50	Top 100	Top $N$
	C3MM [27]	0.268	0.241	0.228	0.345
	NHP [28]	0.265	0.233	0.255	0.323
Positive testing samples with the same number of BiGG reactions	CHESHIRE [29]	0.431	0.383	0.322	0.332
	HGNNP [30]	0.423	0.418	0.382	0.359
	Multi-HGNN [33]	0.501	0.486	0.421	0.434
	**HGLMA**	**0.696 (108/108)**	**0.675 (108/108)**	**0.631 (102/108)**	**0.642 (98/108)**
	NHP [28]	0.023	0.021	0.019	0.024
	CHESHIRE [29]	0.101	0.088	0.078	0.083
Positive testing samples with 16 337 BiGG reactions	HGNNP [30]	0.104	0.095	0.093	0.103
	Multi-HGNN [33]	0.131	0.126	0.112	0.103
	**HGLMA**	**0.208 (101/108)**	**0.205 (97/108)**	**0.200 (102/108)**	**0.172 (98/108)**

Furthermore, we performed the reaction recovery experiments by using candidate reactions formed by positive testing samples and all the 16 337 metabolic reactions. For the cases of Top 25, 50, 100, and $N$, the distributions and average values of recovery rate on 108 BiGG GEMs are presented in Fig. 4e–h and the bottom part of Table 2, respectively. More detailed experiment results of HGLMA can be referred to Supplementary Table S6. Since larger number of candidate reactions increases the difficulty of discovering missing reactions, the overall recovery performances obviously decline compared with those of the above reaction recovery experiments. In spite of this, we can observe from experiment results that the proposed HGLMA significantly improve the performance in terms of recovery rate compared with baseline models, where the average recovery rate values are relatively improved by 58.8%, 62.7%, 78.6%, and 70%, respectively.

As a conclusion, the above results and discussions demonstrate that the proposed HGLMA has the significantly superior capacity of discovering missing reactions from various scales of real metabolic reactions. This can further indicate the greatest potential of HGLMA for GEM gap-fillings.

### Ablation study

To investigate the effectiveness of the metabolite feature extraction module and attention mechanism module of HGLMA, we conducted ablation experiments. By applying five-fold cross-validations on 108 BiGG GEMs, reaction prediction performances of HGLMA and its three variant models were evaluated in terms of AUPRC, recall, F1 score, and accuracy metrics. Three variant models, called HGLMA-v1, HGLMA-v2, and HGLMA-v3, were constructed by properly modifying the metabolite feature extraction module or attention mechanism module of HGLMA. The detailed descriptions are as follows.

- HGLMA-v1: This variant model of HGLMA can be constructed by removing 1D molecular feature extractions of metabolites (ChemBERTa) in the metabolite feature extraction module. HGLMA-v1 only extracts 2D molecular features of metabolites from molecular graph representations.
- HGLMA-v2: This variant model of HGLMA can be constructed by removing the metabolite feature extraction module and using Node2Vec approach [47] to generate initial embeddings of nodes. HGLMA-v2 does not extract any molecular features of metabolites, and the node embeddings are only based on topological properties.
- HGLMA-v3: This variant model of HGLMA can be constructed by removing the attention mechanism module and only using FFN and sigmoid function to calculate hyperedge features as the confidence score from $\hat{\mathbf{x}}_{i}$ for $M_{i} \in \mathcal{V}_{p}$.

**Figure 5.**
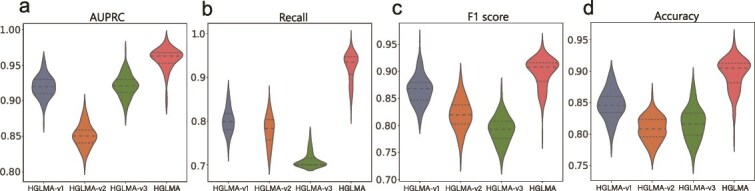
Distributions of prediction performances of HGLMA and its three variant models on 108 BiGG GEMs, in terms of (a) AUPRC, (b) Recall, (c) F1 score, and (d) accuracy.

The distributions and average values of reaction prediction performances of HGLMA and its three variant models on 108 BiGG GEMs are presented in Fig. 5 and Table 3. It is obvious that HGLMA is significantly superior to its three variant models in terms of prediction performance metrics. Compared with five baseline models, these variant models can still achieve better or at least comparable performance metric values.

**Table 3:** Average values of prediction performances of HGLMA and its three variant models on 108 BiGG GEMs

Models	AUPRC	Recall	F1 score	Accuracy
HGLMA-v1	0.920	0.800	0.866	0.847
HGLMA-v2	0.851	0.779	0.820	0.809
HGLMA-v3	0.919	0.711	0.792	0.817
**HGLMA**	**0.959 (100/108)**	**0.924 (108/108)**	**0.897 (89/108)**	**0.895 (100/108)**

The ablation experiment results demonstrate the contributions of the modules introduced to HGLMA for predicting reactions. For instance, HGLMA outperforms HGLMA-v1 by 4.2%, 15.5%, 3.6%, and 5.7%, and HGLMA-v1 is further superior to HGLMA-v2 by 8.1%, 2.7%, 5.6%, and 4.7%, in the average values of AUPRC, recall, F1 score, and accuracy metrics. This indicates that employing modular feature extractions of metabolites as node embeddings is significantly effective to improve prediction performances, and metabolite features can be better mined and exploited through multi-dimensional metabolite feature extractions. Moreover, the relative improvements of HGLMA over HGLMA-v3 in the average values of AUPRC, recall, F1 score, and accuracy metrics are 4.4%, 30%, 13.3%, and 9.6%, respectively. The significant improvement of the recall performance perfectly aligns with the task of discovering missing reactions (recalling positive samples). This demonstrates that it is essential to further identify key metabolite nodes of reactions through the static–dynamic multi-head attention mechanism for the final reaction predictions.

**Figure 6.**
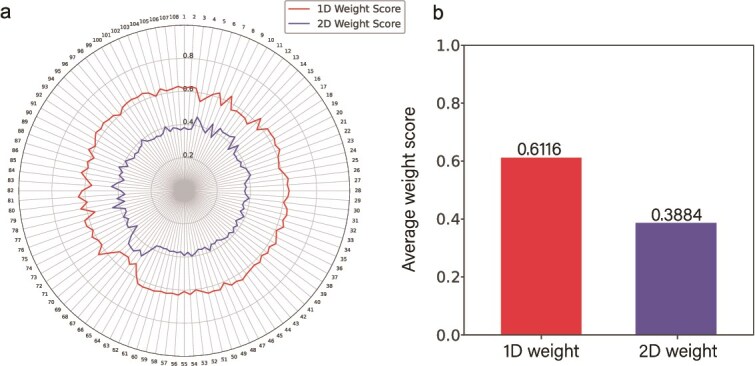
Distributions and average values of normalized 1D and 2D weight scores of HGLMA trained on 108 BiGG GEMs, where the calculation details of normalized 1D and 2D weight scores can be referred to Supplementary Note S2.

In addition, Fig. 6 presents a visualization analysis of the trainable parameters in the embedding matrix for fusing initial feature embeddings of metabolites. This analysis aims to explore the impact of initial embeddings from different dimensions on prediction results. The radar chart shows normalized weight scores of matrix parameters corresponding to multi-dimensional initial metabolite embeddings on each BiGG dataset, and the bar chart displays their normalized average weight scores across all BiGG datasets (see Supplementary Note S2 and Table S7). We note that HGLMA assigns higher weights to 1D feature embeddings (0.6116) than 2D feature embeddings (0.3884). This demonstrates the importance of multi-dimensional feature extractions, and utilizing SMILES sequences of metabolites for feature extractions is effective for reaction predictions.

### Metabolic phenotype prediction

Finally, we conducted metabolic phenotype predictions of fermentation products, in order to test the effectiveness of gap-fillings by HGLMA in the biological meaning. We chose 24 various bacterial organisms, such as *Escherichia coli, Cutibacterium acnes*, etc. Their genomes and experimental fermentation data are available in [17], and the details are also presented in Supplementary Tables S8 and S9. Then, we reconstructed 24 draft GEMs from genomes data of these organisms by using the CarveMe pipeline [16], where the details can be referred to Supplementary Note S3. Following the gap-filling procedure stated as above, we performed training and gap-fillings for each draft GEM individually, and the corresponding gap-filled GEMs by HGLMA were further obtained by filling Top 100 reactions. Following the investigation approach in [29], we used COBRApy [48] to predict the efflux rates (secretion flux divided by biomass) of nine fermentation end products, including acetic acid, formic acid, ethanol, etc. The details of these predicted fermentation products are presented in Supplementary Table S10, where the efflux rates of fermentation products larger than $10^{-5}$ were regarded as produced. By regarding the experimental data of fermentation end products for these 24 organisms as benchmark labels, the phenotype prediction performances of gap-filled GEMs can be evaluated.

**Figure 7.**
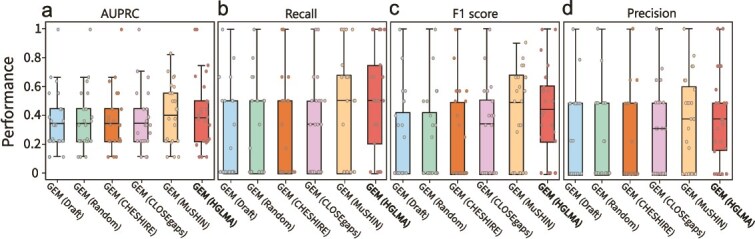
Comparison of metabolic phenotype prediction results among draft GEMs and gap-filled GEMs by random approach, CHESHIRE, CLOSEgaps, MuSHIN, and our proposed HGLMA.

**Figure 8.**
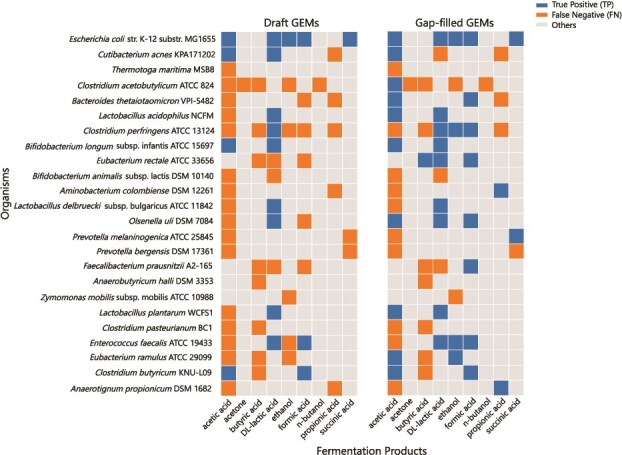
Detailed prediction results of fermentation end products using draft GEMs and the gap-filled GEMs by HGLMA.

For these 24 organisms, Fig. 7 and Table 4, respectively, present the distributions and average values of prediction performances of draft GEMs, GEMs filled by 100 random reactions, and gap-filled GEMs by HGLMA and other three approaches (CHESHIRE, CLOSEgaps, and MuSHIN). The detailed fermentation product prediction results corresponding to our proposed HGLMA are given in Fig. 8 and Supplementary Table S11, and those corresponding to other methods can be referred to Zhao *et al*. [34]. The results demonstrate that the gap-filled GEMs by HGLMA can significantly improve the prediction performances. The average values of AUPRC, recall, F1 score, and precision are relatively increased by 18.5%, 114.9%, 93.2%, and 76% compared with the prediction performance results of draft GEMs, and are relatively increased by 2.9%, 6.1%, 6%, and 5.4% compared with the prediction performance results of gap-filled GEMs by the second best gap-filling method (MuSHIN). Moreover, HGLMA achieves the best prediction performances in terms of AUPRC, recall, F1 score, and precision on 66.7% (16/24), 95.8% (23/24), 75% (18/24), and 79.2% (19/24) of 24 GEMs.

**Table 4:** Comparison of average metabolic phenotype prediction results from different methods

GEMs	AUPRC	Recall	F1 score	Precision
GEM (Draft)	0.356	0.235	0.219	0.221
GEM (Random)	0.356	0.235	0.219	0.221
GEM (CHESHIRE [29])	0.361	0.243	0.229	0.233
GEM (CLOSEgaps [32])	0.364	0.299	0.287	0.288
GEM (MuSHIN [34])	0.410	0.476	0.399	0.369
**GEM (HGLMA)**	**0.422 (16/24)**	**0.505 (23/24)**	**0.423 (18/24)**	**0.389 (19/24)**

For instance, among all fermentation products of 24 draft GEMs, HGLMA can increase the corrected prediction phenotypes of acetic acid from 8 to 13. Moreover, n-butanol, acetone, and propionic acid can be correctly predicted on 95.8% (23/24), 95.8% (23/24), and 79.5% (19/24) of these organisms. With the addition of only the Top 100 reactions, the gap-filled GEMs by HGLMA significantly enhance the consistency between predicted phenotypes and experimental observations, which demonstrates the superior prediction performance for positive samples and the efficient reaction prioritization strategy of the model. The results demonstrate that the GEMs gap-filled by HGLMA exhibit high recall and also a certain number of false positive predictions in phenotypic predictions. Therefore, it can significantly improve the phenotypic prediction performance of the draft GEM with a limited number of additional reactions.

In addition, we have evaluated the corresponding training time and inference time of 24 GEMs. The details of the corresponding training time and inference time are presented in Supplementary Table S12. Therefore, the phenotype prediction results show the usability and superiority of GEM gap-fillings by HGLMA. HGLMA has the potential to serve as a reliable gap-filling tool for reconstructions of high-quality GEMs.

## Conclusion

This study proposed a novel hypergraph learning-based missing reaction prediction approach (HGLMA) for draft GEMs, which combines multi-dimensional metabolite feature extractions and static–dynamic multi-head attention mechanisms. The gap-fillings by this approach can contribute to reconstructing higher-quality GEMs for more accurate phenotype predictions.

The proposed HGLMA consists of the metabolite feature extraction module, hypergraph learning module, and attention mechanism module. In the metabolite feature extraction module, HGLMA simultaneously employs two molecular pretrained language models (ChemBERTa and GraphMVP) to extract multi-dimensional features of metabolite in GEMs and candidate reactions from input SMILES sequences and molecular graphs. These multi-dimensional metabolite features are fused and regarded as initial node embeddings of GEM graphs for further feature extractions. In the hypergraph learning module, we successively use a DGN and a hypergraph neural network (HGNNP) to further extract the features of metabolites and GEMs by mining both directed and high-order metabolic associations within and between reactions. In the attention mechanism module, a static–dynamic multi-head attention mechanism is leveraged to automatically determine attention weights and to identify key metabolites within any candidate reaction based on the extracted features. The confidence score of any candidate metabolite reaction as the missing reaction of the given GEM can be finally predicted.

To evaluate the reaction prediction performances, we conducted five-fold cross-validations on 108 BiGG GEMs, and compared our results with those of other five state-of-the-art baseline methods, which are C3MM, NHP, CHESHIRE, HGNNP, and Multi-HGNN. The results showed that our proposed HGLMA can be significantly superior to other baseline methods for missing reaction predictions. Compared with the second-best approach, the average values of prediction results of HGLMA on 108 GEMs was relatively improved by 5.1%, 22.4%, 15.9%, and 12.6%, in terms of AUPRC, recall, F1 score, and accuracy metrics. Furthermore, we carried out reaction recovery experiments on these 108 GEMs, in order to validate the ability of HGLMA for distinguishing missing reactions from various scales of other reactions. The results also demonstrated the advantages of HGLMA compared with five baseline models. The ablation experiments showed and interpreted the contributions of multi-dimensional metabolite feature extractions and static–dynamic attention mechanisms in HGLMA for missing reaction predictions. In addition, we applied HGLMA to the gap-fillings of 24 draft GEMs of bacterial organisms, and used COBRApy to conduct metabolic phenotype predictions. By regarding the experimental fermentation phenotype data as benchmark labels, the results demonstrated that the gap-filled GEMs by our proposed HGLMA can provide more accurate phenotype predictions. The average values of AUPRC, recall, F1 score, and precision are relatively improved by 18.5%, 114.9%, 93.2%, and 76% compared with those of draft GEMs, and are relatively improved by 2.9%, 6.1%, 6%, and 5.4% compared with those of the second best gap-filling method.

**Key Points**

- This study proposes HGLMA, a novel hypergraph learning approach with multi-dimensional metabolite feature extractions and static–dynamic attention mechanisms, to predict and tease out missing reactions for the gap-fillings of draft GEMs.
- HGLMA simultaneously employs two molecular pretrained language models to conduct multi-dimensional metabolite feature extractions, and uses fused feature representations as node embeddings for the hypergraph learning based on a directional graph network and a hypergraph neural network.
- HGLMA utilizes the static–dynamic multi-head attention mechanism to automatically assign attention weights, so as to identify key metabolites within any candidate metabolic reaction.
- The five-fold cross-validation results on 108 BiGG GEMs show that HGLMA significantly outperforms five state-of-the-art baseline approaches both in prediction performances and in the ability of discovering missing reactions from candidate reaction pools.
- The gap-filled GEMs of 24 organisms by HGLMA were applied for phenotype predictions of nine fermentation products, and the results demonstrate the effectiveness and superiority of gap-fillings by HGLMA, compared with draft GEMs and gap-filled GEMs by other methods.

## Supplementary Material

Supplementary-File_HGLMA_Final_bbag314

## Data Availability

The data and source code of this study are available at https://github.com/kaiwang-group/HGLMA.
