# Supplementary material for: Hypergraph learning with multi-dimensional metabolite feature extractions and static–dynamic attention mechanisms to fill missing reactions in metabolic networks
Source: Brief Bioinform. 2026 Jun 17;27(3):bbag314. doi: 10.1093/bib/bbag314 (PMC13275026; doi:10.1093/bib/bbag314)
Supplement: Supplementary-File_HGLMA_Final_bbag314 [file supplementary-file_hglma_final_bbag314.pdf]

1. Supplementary Notes

Supplementary Note S1: Details of calculating the directed smooth matrices and the directed derivative matrices for the directed graph of GEM.

Consider the directed graph of the GEM as

$$\mathcal{G} = (\mathcal{V}, \mathcal{E})$$

where $\mathcal{V} = \{M_1, M_2, \ldots, M_n\}$ is the set of nodes, and $\mathcal{E} = \{E_1, E_2, .., E_l\}$ represents the set of directed edges. After calculating K smallest eigenvalues of the Laplacian matrix $\mathbf{L}$, which are $\boldsymbol{\varphi}_1, \boldsymbol{\varphi}_2, \ldots, \boldsymbol{\varphi}_K$, we can further obtain the vector field matrix

$$\mathbf{F}_k = \nabla \boldsymbol{\varphi}_k \in \mathbb{R}^{n \times n}$$

where

$$[\nabla \boldsymbol{\varphi}_k]_{i,j} = \begin{cases} [\boldsymbol{\varphi}_k]_i - [\boldsymbol{\varphi}_k]_j, & M_i \text{ and } M_j \text{ is connected} \\ 0, & M_i \text{ and } M_j \text{ is unconnected} \end{cases}$$

$[\bullet]_{i,j}$ denotes the (i,j)-th entry of the matrix $\bullet$, and $[\bullet]_i$ denotes the i-th entry of the vector $\bullet$.

Furthermore, we can obtain the directed smoothing matrix

$$\mathbf{B}_k^{\mathrm{av}} = \left| \hat{\mathbf{F}}_k \right|$$

and the directed derivative matrix $\mathbf{B}_k^{\mathrm{dx}} \in \mathbb{R}^{n \times n}$ as

$$\mathbf{B}_k^{\mathrm{dx}} = \hat{\mathbf{F}}_k - \mathrm{diag}\left(\sum_{j=1}^{n} \left[\hat{\mathbf{F}}_k\right]_{1,j}, \sum_{j=1}^{n} \left[\hat{\mathbf{F}}_k\right]_{2,j}, \ldots, \sum_{j=1}^{n} \left[\hat{\mathbf{F}}_k\right]_{n,j} \right)$$

where $k = 1, 2, ..., K$. Moreover, $\hat{\mathbf{F}}_k \in \mathbb{R}^{n \times n}$ satisfy

$$\left[\hat{\mathbf{F}}_k\right]_{i,:} = \frac{[\mathbf{F}_k]_{i,:}}{\left\| [\mathbf{F}_k]_{i,:}^{\mathrm{T}} \right\|_1 + \epsilon}$$

where $i = 1, 2, ..., n$, $\varepsilon > 0$ is a sufficiently small positive constant, $[\bullet]_{i,:}$ denotes the i-th row (vector) of the matrix $\bullet$, and $\|\bullet\|_1$ denotes the 1-norm of the vector $\bullet$.

Supplementary Note S2: Calculations of normalized one-dimensional and two-dimensional weight scores.

The normalized one-dimensional (1D) weight score is calculated by

$$\text{normalized 1D weight score} = \frac{\dfrac{\sum_{i=1}^{N_1} \left\| [\mathbf{W}_1]_{:,i} \right\|_1}{N_1}}{\dfrac{\sum_{i=1}^{N_1} \left\| [\mathbf{W}_1]_{:,i} \right\|_1}{N_1} + \dfrac{\sum_{j=N_1+1}^{N_1+N_2} \left\| [\mathbf{W}_1]_{:,j} \right\|_1}{N_2}}$$

and the normalized two-dimensional (2D) weight score is calculated by

$$\text{normalized 2D weight score} = \frac{\dfrac{\sum_{j=N_1+1}^{N_1+N_2} \left\| [\mathbf{W}_1]_{:,j} \right\|_1}{N_2}}{\dfrac{\sum_{i=1}^{N_1} \left\| [\mathbf{W}_1]_{:,i} \right\|_1}{N_1} + \dfrac{\sum_{j=N_1+1}^{N_1+N_2} \left\| [\mathbf{W}_1]_{:,j} \right\|_1}{N_2}}$$

where N_1 is the dimension of 2D feature representation vectors, N_2 is the dimension of 2D feature representation vectors, $[\mathbf{W}_1]_{:,i}$ and $[\mathbf{W}_1]_{:,j}$ denote the *i*-th and *j*-th columns of $\mathbf{W}_1 \in \mathbb{R}^{N_3 \times (N_1+N_2)}$ respectively, and $\left\| \bullet \right\|_1$ denotes the 1-norm of the vector $\bullet$.

Supplementary Note S3: Details of generating 24 draft GEMs in metabolic phenotype predictions.

According to CHESHIRE [29], the draft GEMs were reconstructed using CarveMe [16]. First, the genome sequences in FASTA file formats of the 24 organisms in Section "Metabolic phenotype prediction" were searched and downloaded from the NCBI Assembly database website (https://www.ncbi.nlm.nih.gov), according to the information of these 24 organisms listed in Supplementary Table S8. Then, by inputting these genome sequences, we employed the CarveMe reconstruction tool, which is based on the top-down GEM reconstruction strategy and is combined with the DIAMOND sequence alignment and mixed-integer linear programming (MILP) method, to obtain the corresponding 24 draft GEMs in xml format. The CarveMe tool is available online (https://github.com/cdanielmachado/carveme). Finally, these 24 draft GEMs in xml format were uploaded online (https://github.com/kaiwang-group/HGLMA), which can be further used for gap-fillings and metabolic phenotype predictions.

2. Supplementary Figures

Supplementary Figure S1: GEM reconstruction workflows of successively using automated reconstruction tools and gap-fillings by HGLMA.

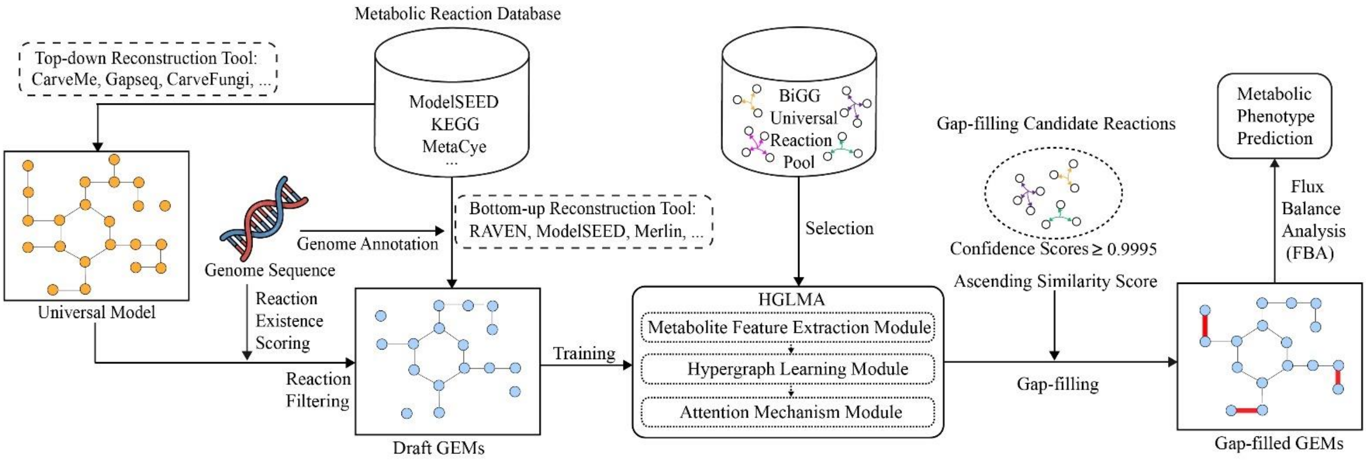

Supplementary Figure S2: Hyperparameter analysis of HGLMA on iBWG-1329. (A) Learning rate. (B) Number of smallest eigenvalues of the Laplacian matrix *K*. (C) Number of layers (DGN) L_1. (D) Number of layers (HGNNP) L_2.

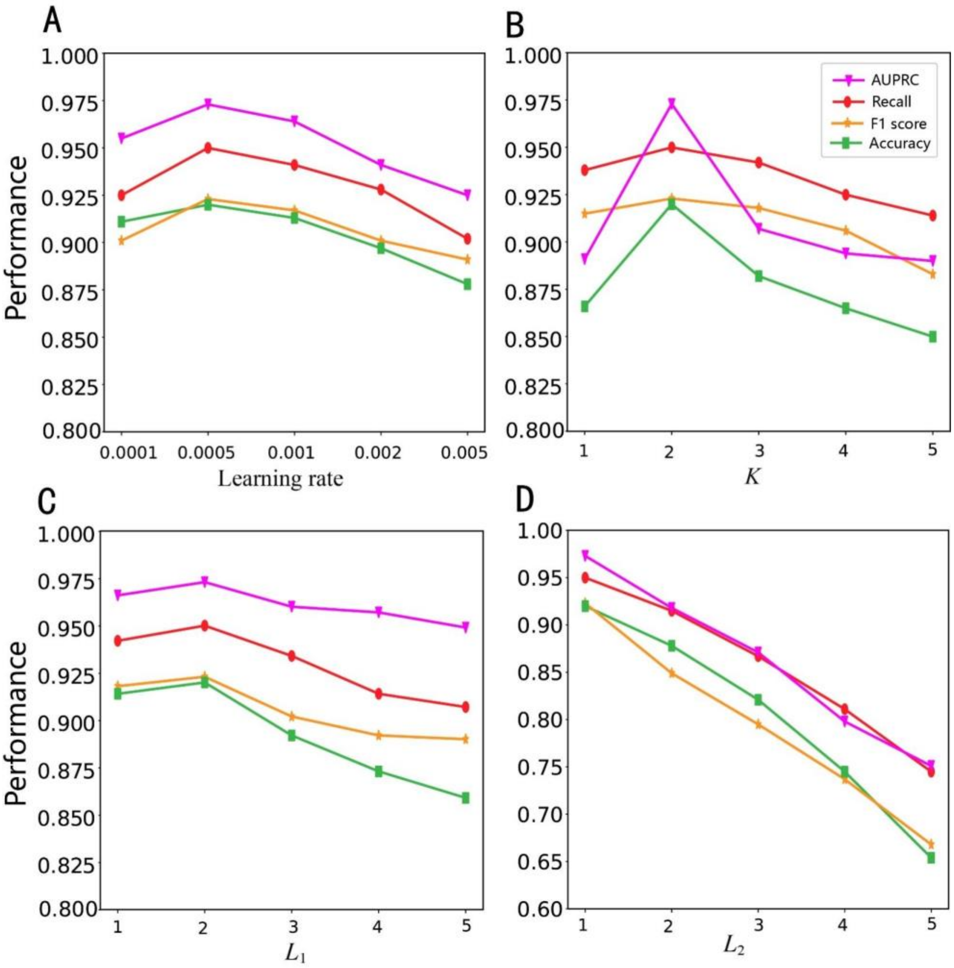

Supplementary Figure S3: Reaction prediction performance metrics of HGLMA on (A) iAF1260b and (B) Recon3D through different negative sampling methods, where the real negative samples were randomly selected from the BiGG universal reaction pool with the same number as that of positive samples.

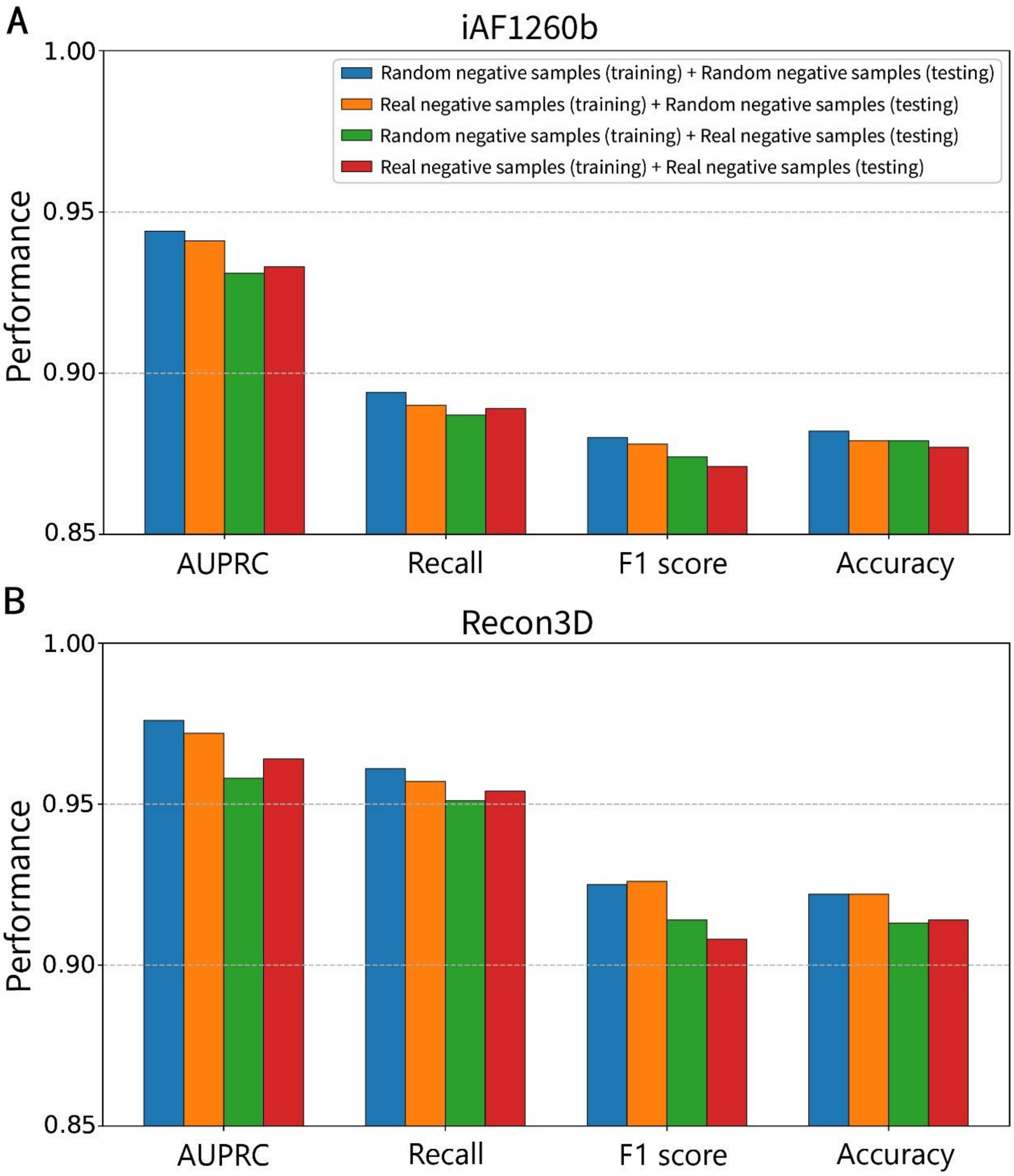

3. Supplementary Tables

Supplementary Table S1: Details of learnable parameter matrices/vectors in HGLMA.

Modules	Learnable parameter matrices/vectors
Metabolite feature extraction module: FFN	$\mathbf{W}_1 \in \mathbb{R}^{N_3 \times (N_1+N_2)}$, $\mathbf{b}_1 \in \mathbb{R}^{N_3}$
Hypergraph learning module: DGN	$\mathbf{W}_2^{(t)} \in \mathbb{R}^{N_3 \times N_3}$, $\mathbf{b}_2^{(t)} \in \mathbb{R}^{N_3}$, $t = 0,1,\ldots,L_1-1$<br>$\mathbf{W}_2^{(L_1)} \in \mathbb{R}^{N_4 \times N_3}$, $\mathbf{b}_2^{(L_1)} \in \mathbb{R}^{N_4}$
Hypergraph learning module: HGNNP	$\mathbf{W}_3^{(t)} \in \mathbb{R}^{N_4 \times N_4}$, $\mathbf{W}_3^{(L_2)} \in \mathbb{R}^{N_4 \times N_5}$, $t = 0,1,\ldots,L_2-1$
Attention mechanism module: static-dynamic attention mechanism	$\mathbf{W}_\mathrm{S} \in \mathbb{R}^{N_5 \times N_5}$
	$\mathbf{W}_\mathrm{Q}^{(k)}, \mathbf{W}_\mathrm{K}^{(k)} \in \mathbb{R}^{N_5 \times (n_\mathrm{head} N_6)}$, $\mathbf{W}_\mathrm{V}^{(k)} \in \mathbb{R}^{N_5 \times (n_\mathrm{head} N_6)}$
	$\mathbf{W}_\mathrm{O} \in \mathbb{R}^{N_5 \times (n_\mathrm{head} N_7)}$
Attention mechanism module: FFN	$\mathbf{W}_4 \in \mathbb{R}^{1 \times N_5}$, $\mathbf{b}_4 \in \mathbb{R}$

Supplementary Table S2: Hyperparameter values of constructing and training HGLMA.

Hyperparameters	Values
1D metabolite feature representation dimension N_1	768
2D metabolite feature representation dimension N_2	2048
Initial feature embedding dimension N_3	512
Feature representation dimension (DGN) N_4	256
Feature representation dimension (HGNNP) N_5	64
Hyperparameters in attention mechanism module N_6, N_7, n_{head}	16, 16, 8
Number of smallest eigenvalues of the Laplacian matrix K	2
Number of layers (DGN) L_1	2
Number of layers (HGNNP) L_2	1
Initial learning rate	0.0005
Batch sizes for different GEMs n_{batch}	$16 \leq n_{\text{batch}} \leq 96$
Epoch	500
Dropout rate in HGNNP	0.5
Dropout rate in static-dynamic attention mechanism	0.3
Dropout rate in the final FFN	0.4

Supplementary Table S3: Detailed 5-fold cross-validation results of reaction prediction performances of HGLMA on 108 BiGG GEMs.

	fold0				fold1				fold2			
GEMs	AUPRC	Recall	F1 score	Accuracy	AUPRC	Recall	F1 score	Accuracy	AUPRC	Recall	F1 score	Accuracy
iAF1260b	0.915	0.69	0.791	0.818	0.953	0.932	0.905	0.902	0.947	0.951	0.889	0.882
iAF692	0.905	0.892	0.901	0.828	0.89	0.877	0.916	0.828	0.92	0.907	0.886	0.827
iAF1260	0.95	0.958	0.872	0.859	0.964	0.951	0.909	0.905	0.971	0.969	0.92	0.915
iAF987	0.988	0.95	0.922	0.917	0.977	0.964	0.879	0.867	0.948	0.969	0.888	0.878
iAM_Pb448	0.904	0.857	0.85	0.855	0.889	0.842	0.865	0.857	0.919	0.872	0.835	0.854
iAM_Pc455	0.945	0.846	0.841	0.861	0.96	0.861	0.826	0.863	0.93	0.831	0.856	0.863
iAM_Pf480	0.94	0.86	0.851	0.872	0.925	0.845	0.866	0.874	0.955	0.875	0.836	0.871
iAM_Pk459	0.948	0.876	0.846	0.861	0.933	0.861	0.861	0.863	0.963	0.891	0.831	0.863
iAM_Pv461	0.947	0.88	0.864	0.872	0.932	0.895	0.849	0.874	0.962	0.865	0.879	0.871
iAPECO1_1312	0.951	0.874	0.854	0.866	0.936	0.859	0.869	0.868	0.966	0.889	0.839	0.865
iAT_PLT_636	0.959	0.896	0.903	0.904	0.971	0.927	0.894	0.891	0.983	0.938	0.928	0.927
iBWG_1329	0.971	0.924	0.921	0.915	0.986	0.939	0.906	0.917	0.956	0.909	0.936	0.915
iB21_1397	0.97	0.926	0.924	0.916	0.955	0.911	0.939	0.914	0.985	0.941	0.909	0.916
iCHOv1	0.986	0.975	0.949	0.948	0.976	0.991	0.922	0.917	0.992	0.964	0.956	0.956
iCHOv1_DG44	0.984	0.935	0.928	0.927	0.959	0.967	0.906	0.900	0.978	0.949	0.924	0.922
iCN900	0.966	0.958	0.906	0.901	0.988	0.948	0.953	0.953	0.982	0.938	0.945	0.945
ic_1306	0.957	0.979	0.9	0.892	0.981	0.954	0.935	0.933	0.98	0.927	0.919	0.919
iE2348C_1286	0.954	0.958	0.914	0.909	0.948	0.915	0.907	0.906	0.876	0.738	0.808	0.825
iEC042_1314	0.915	0.738	0.815	0.832	0.972	0.931	0.909	0.906	0.931	0.927	0.898	0.895
iEC1344_C	0.95	0.887	0.887	0.886	0.94	0.958	0.895	0.888	0.987	0.973	0.948	0.947
iEC1349_Crooks	0.96	0.967	0.9	0.893	0.942	0.954	0.887	0.878	0.989	0.958	0.949	0.949
iEC1356_Bl21DE3	0.95	0.9	0.893	0.893	0.978	0.927	0.924	0.924	0.961	0.946	0.905	0.901
iEC1364_W	0.939	0.938	0.889	0.883	0.959	0.904	0.901	0.901	0.98	0.923	0.927	0.927
iEC1368_DH5a	0.98	0.944	0.927	0.926	0.966	0.973	0.893	0.883	0.954	0.979	0.905	0.897
iEC1372_W3110	0.954	0.971	0.89	0.880	0.962	0.91	0.912	0.913	0.942	0.929	0.89	0.885
iEC55989_1330	0.985	0.965	0.951	0.950	0.966	0.975	0.916	0.910	0.969	0.94	0.908	0.905
iECABU_c1320	0.969	0.967	0.899	0.892	0.973	0.894	0.907	0.908	0.982	0.952	0.944	0.944
iECBD_1354	0.967	0.908	0.906	0.906	0.913	0.752	0.813	0.827	0.95	0.942	0.894	0.889

	fold0				fold1				fold2			
GEMs	AUPRC	Recall	F1 score	Accuracy	AUPRC	Recall	F1 score	Accuracy	AUPRC	Recall	F1 score	Accuracy
iECB_1328	0.98	0.977	0.936	0.933	0.965	0.95	0.914	0.910	0.984	0.931	0.935	0.935
iECDH10B_1368	0.983	0.965	0.929	0.926	0.951	0.944	0.883	0.875	0.941	0.894	0.87	0.867
iECDH1ME8569_1439	0.986	0.971	0.94	0.939	0.944	0.925	0.892	0.888	0.959	0.958	0.925	0.922
iEcDH1_1363	0.955	0.908	0.888	0.885	0.981	0.969	0.954	0.953	0.984	0.948	0.933	0.932
iECD_1391	0.975	0.963	0.912	0.907	0.966	0.921	0.907	0.905	0.967	0.906	0.911	0.911
iEcE24377_1341	0.959	0.956	0.905	0.900	0.982	0.965	0.941	0.940	0.93	0.931	0.891	0.886
iECED1_1282	0.98	0.963	0.94	0.939	0.979	0.965	0.928	0.925	0.934	0.952	0.92	0.917
iECH74115_1262	0.965	0.952	0.911	0.907	0.98	0.963	0.942	0.941	0.968	0.944	0.92	0.918
iEcHS_1320	0.965	0.954	0.893	0.885	0.973	0.952	0.906	0.901	0.959	0.967	0.882	0.871
iECIAI1_1343	0.912	0.688	0.787	0.814	0.975	0.965	0.928	0.925	0.942	0.933	0.904	0.901
iECIAI39_1322	0.953	0.946	0.887	0.879	0.984	0.973	0.952	0.951	0.927	0.892	0.883	0.882
iECNA114_1301	0.982	0.956	0.943	0.943	0.959	0.969	0.913	0.907	0.951	0.935	0.905	0.902
iECO103_1326	0.982	0.952	0.945	0.945	0.967	0.923	0.905	0.903	0.955	0.958	0.904	0.898
iECO111_1330	0.961	0.944	0.907	0.903	0.947	0.94	0.89	0.884	0.973	0.927	0.917	0.916
iECO26_1355	0.958	0.923	0.88	0.874	0.977	0.977	0.931	0.928	0.982	0.952	0.939	0.939
iECOK1_1307	0.943	0.944	0.897	0.892	0.984	0.956	0.94	0.939	0.96	0.946	0.898	0.893
iEcolC_1368	0.936	0.912	0.871	0.865	0.955	0.954	0.896	0.890	0.983	0.942	0.931	0.930
iECP_1309	0.976	0.912	0.919	0.920	0.952	0.929	0.881	0.875	0.978	0.931	0.921	0.920
iECS88_1305	0.93	0.827	0.857	0.861	0.961	0.985	0.901	0.892	0.965	0.94	0.907	0.903
iECSE_1348	0.983	0.969	0.943	0.942	0.945	0.935	0.892	0.886	0.956	0.94	0.9	0.896
iECSF_1327	0.937	0.95	0.887	0.879	0.97	0.915	0.915	0.915	0.981	0.958	0.931	0.929
iEcSMS35_1347	0.943	0.942	0.882	0.874	0.978	0.96	0.927	0.924	0.976	0.946	0.911	0.907
iECSP_1301	0.955	0.946	0.882	0.873	0.96	0.938	0.896	0.892	0.979	0.975	0.932	0.929
iECs_1301	0.98	0.929	0.917	0.916	0.979	0.958	0.931	0.929	0.965	0.921	0.907	0.905
iECUMN_1333	0.966	0.931	0.91	0.908	0.921	0.912	0.88	0.875	0.957	0.938	0.905	0.902
iECW_1372	0.986	0.929	0.935	0.935	0.956	0.94	0.899	0.895	0.954	0.935	0.894	0.890
iEK1008	0.976	0.969	0.905	0.898	0.968	0.964	0.898	0.891	0.985	0.969	0.932	0.930
iEKO11_1354	0.98	0.944	0.925	0.924	0.957	0.94	0.906	0.902	0.985	0.95	0.934	0.933
iETEC_1333	0.985	0.956	0.943	0.943	0.981	0.958	0.936	0.934	0.968	0.95	0.919	0.917
iG2583_1286	0.983	0.95	0.943	0.943	0.958	0.952	0.897	0.891	0.949	0.94	0.908	0.905
iHN637	0.966	0.896	0.92	0.922	0.957	0.885	0.895	0.896	0.955	0.854	0.863	0.865

	fold0				fold1				fold2			
GEMs	AUPRC	Recall	F1 score	Accuracy	AUPRC	Recall	F1 score	Accuracy	AUPRC	Recall	F1 score	Accuracy
iIS312	0.975	0.948	0.929	0.927	0.955	0.938	0.887	0.880	0.949	0.896	0.864	0.859
iIS312_Amastigote	0.971	0.969	0.89	0.880	0.965	0.969	0.925	0.922	0.947	0.865	0.86	0.859
iIS312_Epimastigote	0.928	0.865	0.869	0.870	0.963	0.927	0.886	0.880	0.974	0.958	0.953	0.953
iIS312_Trypomastigote	0.854	0.438	0.583	0.688	0.972	0.99	0.927	0.922	0.98	0.969	0.939	0.938
iIT341	0.956	0.906	0.849	0.839	0.93	0.823	0.827	0.828	0.893	0.896	0.819	0.802
iJB785	0.965	0.948	0.901	0.896	0.991	0.979	0.94	0.938	0.982	0.938	0.952	0.953
iJN1463	0.942	0.951	0.89	0.883	0.948	0.925	0.876	0.869	0.95	0.931	0.871	0.862
iJN678	0.973	1	0.842	0.813	0.983	0.885	0.924	0.927	0.976	0.885	0.904	0.906
iJN746	0.906	0.891	0.82	0.805	0.906	0.932	0.836	0.818	0.842	0.375	0.524	0.659
iJO1366	0.965	0.938	0.896	0.891	0.981	0.946	0.935	0.934	0.98	0.963	0.934	0.932
iJR904	0.919	0.786	0.844	0.854	0.949	0.964	0.845	0.823	0.983	0.958	0.92	0.917
iLB1027_lipid	0.987	0.998	0.909	0.900	0.933	0.26	0.411	0.627	0.996	1	0.91	0.901
iLF82_1304	0.936	0.944	0.865	0.853	0.963	0.935	0.923	0.922	0.983	0.956	0.937	0.935
iLJ478	0.942	0.927	0.868	0.859	0.969	0.948	0.91	0.906	0.946	0.969	0.853	0.833
iML1515	0.984	0.956	0.94	0.939	0.969	0.963	0.93	0.927	0.952	0.952	0.88	0.870
iMM1415	0.971	0.96	0.925	0.923	0.972	0.935	0.913	0.911	0.926	0.814	0.852	0.859
iMM904	0.969	0.979	0.901	0.892	0.952	0.958	0.852	0.833	0.973	0.965	0.924	0.920
iND750	0.981	0.979	0.893	0.883	0.94	0.953	0.874	0.862	0.92	0.781	0.831	0.841
iNF517	0.983	0.938	0.942	0.943	0.987	0.948	0.929	0.927	0.977	0.948	0.919	0.917
iNJ661	0.961	0.927	0.925	0.924	0.97	0.875	0.903	0.906	0.945	0.948	0.839	0.818
iNRG857_1313	0.986	0.963	0.947	0.946	0.963	0.971	0.902	0.895	0.953	0.931	0.898	0.894
iPC815	0.967	0.922	0.91	0.909	0.931	0.94	0.877	0.868	0.935	0.938	0.885	0.878
iRC1080	0.979	0.917	0.924	0.924	0.97	0.992	0.878	0.862	0.991	0.958	0.956	0.956
iSB619	0.969	0.969	0.939	0.938	0.972	0.917	0.907	0.906	0.976	0.927	0.927	0.927
iSbBS512_1146	0.959	0.948	0.904	0.899	0.956	0.906	0.899	0.898	0.975	0.963	0.928	0.925
iSBO_1134	0.962	0.952	0.916	0.913	0.982	0.925	0.931	0.931	0.934	0.883	0.876	0.875
iSDY_1059	0.947	0.912	0.872	0.866	0.948	0.965	0.892	0.883	0.857	0.617	0.728	0.770
iSFV_1184	0.985	0.948	0.935	0.934	0.948	0.919	0.892	0.889	0.968	0.944	0.918	0.916
iSFxv_1172	0.96	0.981	0.907	0.899	0.976	0.927	0.935	0.935	0.947	0.925	0.898	0.895
iSF_1195	0.906	0.946	0.88	0.871	0.94	0.95	0.891	0.883	0.982	0.94	0.936	0.935
iSSON_1240	0.984	0.969	0.945	0.944	0.964	0.96	0.923	0.920	0.958	0.923	0.907	0.905

	fold0				fold1				fold2			
GEMs	AUPRC	Recall	F1 score	Accuracy	AUPRC	Recall	F1 score	Accuracy	AUPRC	Recall	F1 score	Accuracy
iS_1188	0.937	0.94	0.876	0.867	0.938	0.898	0.869	0.865	0.978	0.915	0.926	0.927
iUMN146_1321	0.966	0.915	0.912	0.911	0.98	0.931	0.925	0.924	0.984	0.942	0.94	0.940
iUMNK88_1353	0.985	0.963	0.931	0.929	0.937	0.929	0.882	0.876	0.963	0.965	0.911	0.906
iUTI89_1310	0.969	0.931	0.913	0.911	0.969	0.971	0.916	0.910	0.972	0.948	0.924	0.922
iWFL_1372	0.946	0.946	0.895	0.890	0.985	0.958	0.938	0.936	0.986	0.946	0.949	0.949
iY75_1357	0.962	0.956	0.922	0.919	0.982	0.931	0.927	0.927	0.985	0.948	0.94	0.940
iYL1228	0.966	0.961	0.903	0.897	0.935	0.922	0.874	0.867	0.924	0.615	0.739	0.783
iYO844	0.963	0.948	0.883	0.875	0.979	0.974	0.901	0.893	0.921	0.74	0.816	0.833
iYS1720	0.982	0.924	0.928	0.929	0.968	0.954	0.928	0.926	0.979	0.946	0.938	0.938
iYS854	0.959	0.931	0.887	0.882	0.947	0.899	0.895	0.894	0.969	0.955	0.923	0.920
iZ_1308	0.979	0.927	0.92	0.920	0.964	0.935	0.893	0.888	0.943	0.925	0.883	0.877
RECON1	0.987	0.96	0.945	0.944	0.983	0.942	0.936	0.935	0.987	0.948	0.946	0.946
Recon3D	0.985	0.95	0.939	0.938	0.982	0.958	0.93	0.928	0.961	0.963	0.897	0.889
STM_v1_0	0.978	0.925	0.927	0.927	0.955	0.933	0.892	0.886	0.945	0.835	0.868	0.873
iCN718	0.96	0.932	0.913	0.911	0.975	0.947	0.898	0.911	0.945	0.917	0.928	0.912
iAB_RBC_283	0.971	0.922	0.901	0.898	0.932	0.797	0.829	0.836	0.952	0.875	0.862	0.859
iSynCJ816	0.927	0.927	0.868	0.859	0.93	0.958	0.814	0.781	0.932	0.912	0.883	0.860
e_coli_core	0.826	0.938	0.833	0.813	0.821	0.812	0.867	0.875	0.955	1	0.914	0.906

	fold3				fold4				all-fold			
GEMs	AUPRC	Recall	F1 score	Accuracy	AUPRC	Recall	F1 score	Accuracy	AUPRC	Recall	F1 score	Accuracy
iAF1260b	0.969	0.951	0.911	0.908	0.937	0.948	0.905	0.901	0.944	0.894	0.88	0.882
iAF692	0.89	0.877	0.916	0.827	0.92	0.907	0.886	0.828	0.905	0.892	0.901	0.828
iAF1260	0.965	0.961	0.89	0.882	0.98	0.946	0.875	0.883	0.966	0.857	0.893	0.889
iAF987	0.97	0.964	0.905	0.898	0.907	0.505	0.66	0.740	0.958	0.878	0.851	0.860
iAM_Pb448	0.889	0.842	0.865	0.856	0.889	0.872	0.835	0.855	0.898	0.857	0.85	0.855
iAM_Pc455	0.96	0.861	0.826	0.862	0.93	0.831	0.856	0.862	0.945	0.846	0.841	0.862
iAM_Pf480	0.925	0.845	0.866	0.873	0.955	0.875	0.836	0.872	0.94	0.86	0.851	0.872
iAM_Pk459	0.933	0.861	0.861	0.862	0.963	0.891	0.831	0.862	0.948	0.876	0.846	0.862
iAM_Pv461	0.932	0.895	0.849	0.873	0.962	0.865	0.879	0.872	0.947	0.88	0.864	0.873
iAPECO1_1312	0.936	0.859	0.869	0.867	0.966	0.889	0.839	0.865	0.951	0.874	0.854	0.866
iAT_PLT_636	0.968	0.849	0.886	0.891	0.983	0.911	0.921	0.922	0.973	0.904	0.906	0.907
iBWG_1329	0.956	0.939	0.906	0.916	0.956	0.909	0.936	0.916	0.967	0.925	0.922	0.916
iB21_1397	0.955	0.911	0.939	0.915	0.975	0.941	0.909	0.915	0.961	0.926	0.924	0.915
iCHOv1	0.978	0.993	0.941	0.938	0.995	0.985	0.972	0.972	0.986	0.982	0.948	0.946
iCHOv1_DG44	0.982	0.943	0.93	0.929	0.973	0.953	0.93	0.928	0.975	0.949	0.924	0.921
iCN900	0.979	0.964	0.914	0.909	0.965	0.953	0.904	0.898	0.976	0.952	0.924	0.921
ic_1306	0.959	0.944	0.903	0.899	0.966	0.942	0.92	0.918	0.968	0.949	0.915	0.912
iE2348C_1286	0.929	0.971	0.899	0.891	0.957	0.942	0.906	0.902	0.933	0.905	0.887	0.887
iEC042_1314	0.959	0.967	0.897	0.890	0.945	0.869	0.886	0.889	0.945	0.886	0.881	0.882
iEC1344_C	0.977	0.95	0.945	0.945	0.928	0.954	0.884	0.875	0.956	0.945	0.912	0.908
iEC1349_Crooks	0.986	0.952	0.937	0.936	0.957	0.977	0.882	0.869	0.967	0.962	0.911	0.905
iEC1356_Bl21DE3	0.967	0.963	0.921	0.918	0.986	0.958	0.94	0.939	0.968	0.939	0.917	0.915
iEC1364_W	0.981	0.94	0.93	0.929	0.962	0.933	0.907	0.904	0.964	0.927	0.911	0.909
iEC1368_DH5a	0.986	0.95	0.946	0.946	0.966	0.948	0.913	0.909	0.971	0.959	0.917	0.912
iEC1372_W3110	0.974	0.971	0.923	0.919	0.967	0.969	0.897	0.889	0.96	0.95	0.902	0.897
iEC55989_1330	0.945	0.942	0.881	0.873	0.983	0.956	0.937	0.935	0.97	0.955	0.919	0.915
iECABU_c1320	0.973	0.921	0.913	0.913	0.97	0.96	0.92	0.917	0.973	0.939	0.917	0.915
iECBD_1354	0.955	0.952	0.889	0.881	0.956	0.954	0.888	0.879	0.948	0.902	0.878	0.876
iECB_1328	0.944	0.956	0.873	0.861	0.963	0.946	0.904	0.900	0.967	0.952	0.913	0.908
iECDH10B_1368	0.955	0.971	0.924	0.920	0.985	0.952	0.942	0.942	0.963	0.945	0.91	0.906

	fold3				fold4				all-fold			
GEMs	AUPRC	Recall	F1 score	Accuracy	AUPRC	Recall	F1 score	Accuracy	AUPRC	Recall	F1 score	Accuracy
iECDH1ME8569_1439	0.975	0.925	0.925	0.925	0.967	0.956	0.907	0.902	0.966	0.947	0.918	0.915
iEcDH1_1363	0.964	0.954	0.901	0.895	0.972	0.956	0.917	0.914	0.971	0.947	0.919	0.916
iECD_1391	0.952	0.956	0.886	0.877	0.983	0.956	0.938	0.936	0.969	0.94	0.911	0.908
iEcE24377_1341	0.96	0.958	0.91	0.905	0.985	0.969	0.948	0.947	0.963	0.956	0.919	0.916
iECED1_1282	0.968	0.938	0.898	0.894	0.94	0.86	0.888	0.892	0.96	0.935	0.915	0.913
iECH74115_1262	0.95	0.938	0.892	0.886	0.957	0.952	0.919	0.916	0.964	0.95	0.917	0.914
iEcHS_1320	0.98	0.956	0.937	0.935	0.986	0.948	0.946	0.946	0.973	0.955	0.913	0.908
iECIAI1_1343	0.916	0.944	0.895	0.890	0.928	0.887	0.887	0.886	0.935	0.883	0.88	0.883
iECIAI39_1322	0.948	0.925	0.894	0.891	0.98	0.96	0.941	0.940	0.958	0.939	0.911	0.909
iECNA114_1301	0.973	0.944	0.932	0.931	0.965	0.942	0.906	0.902	0.966	0.949	0.92	0.917
iECO103_1326	0.975	0.952	0.935	0.933	0.953	0.927	0.904	0.902	0.967	0.943	0.919	0.916
iECO111_1330	0.976	0.931	0.931	0.931	0.945	0.933	0.901	0.897	0.961	0.935	0.909	0.906
iECO26_1355	0.946	0.95	0.891	0.883	0.966	0.921	0.888	0.883	0.966	0.945	0.906	0.901
iECOK1_1307	0.965	0.95	0.899	0.894	0.986	0.944	0.937	0.936	0.968	0.948	0.914	0.911
iEcolC_1368	0.989	0.969	0.954	0.953	0.956	0.883	0.887	0.888	0.964	0.932	0.908	0.905
iECP_1309	0.944	0.94	0.877	0.869	0.954	0.931	0.911	0.909	0.961	0.929	0.902	0.899
iECS88_1305	0.961	0.931	0.901	0.898	0.96	0.929	0.91	0.908	0.955	0.922	0.895	0.892
iECSE_1348	0.95	0.967	0.898	0.891	0.99	0.975	0.944	0.942	0.965	0.957	0.915	0.911
iECSF_1327	0.953	0.931	0.875	0.867	0.955	0.958	0.913	0.908	0.959	0.942	0.904	0.900
iEcSMS35_1347	0.968	0.89	0.905	0.906	0.963	0.979	0.907	0.900	0.966	0.943	0.906	0.902
iECSP_1301	0.96	0.925	0.916	0.916	0.985	0.931	0.94	0.941	0.968	0.943	0.913	0.910
iECs_1301	0.94	0.927	0.902	0.899	0.947	0.935	0.899	0.895	0.962	0.934	0.911	0.909
iECUMN_1333	0.937	0.91	0.87	0.864	0.986	0.96	0.945	0.944	0.953	0.93	0.902	0.899
iECW_1372	0.942	0.946	0.889	0.882	0.974	0.95	0.927	0.925	0.962	0.94	0.909	0.905
iEK1008	0.959	0.719	0.831	0.854	0.957	0.953	0.863	0.849	0.969	0.915	0.886	0.884
iEKO11_1354	0.965	0.956	0.894	0.886	0.97	0.948	0.923	0.921	0.971	0.948	0.916	0.913
iETEC_1333	0.952	0.946	0.924	0.922	0.931	0.946	0.878	0.869	0.963	0.951	0.92	0.917
iG2583_1286	0.943	0.938	0.89	0.884	0.982	0.954	0.933	0.931	0.963	0.947	0.914	0.911
iHN637	0.964	0.906	0.916	0.917	0.965	0.969	0.877	0.865	0.961	0.902	0.894	0.893
iIS312	0.969	0.927	0.904	0.901	0.969	0.792	0.869	0.880	0.963	0.9	0.89	0.890
iIS312_Amastigote	0.967	0.917	0.903	0.901	0.931	0.594	0.74	0.792	0.956	0.863	0.864	0.871

	fold3				fold4				all-fold			
GEMs	AUPRC	Recall	F1 score	Accuracy	AUPRC	Recall	F1 score	Accuracy	AUPRC	Recall	F1 score	Accuracy
iIS312_Epimastigote	0.911	0.927	0.894	0.891	0.936	0.927	0.873	0.865	0.942	0.921	0.895	0.892
iIS312_Trypomastigote	0.992	0.99	0.945	0.943	0.949	0.896	0.864	0.859	0.949	0.856	0.852	0.870
iIT341	0.945	0.906	0.849	0.839	0.945	0.792	0.844	0.854	0.934	0.865	0.838	0.832
iJB785	0.966	0.927	0.908	0.906	0.975	0.917	0.917	0.917	0.976	0.942	0.924	0.922
iJN1463	0.888	0.66	0.752	0.783	0.965	0.905	0.895	0.894	0.939	0.874	0.857	0.858
iJN678	0.951	0.99	0.888	0.875	0.97	0.958	0.893	0.885	0.97	0.944	0.89	0.881
iJN746	0.935	0.948	0.845	0.826	0.96	0.953	0.869	0.857	0.91	0.82	0.779	0.793
iJO1366	0.964	0.971	0.904	0.897	0.962	0.95	0.899	0.894	0.971	0.953	0.914	0.910
iJR904	0.958	0.938	0.874	0.865	0.952	0.927	0.834	0.815	0.952	0.915	0.863	0.855
iLB1027_lipid	0.994	0.992	0.939	0.935	0.988	0.998	0.943	0.939	0.98	0.85	0.822	0.861
iLF82_1304	0.982	0.946	0.929	0.928	0.949	0.9	0.887	0.885	0.963	0.936	0.908	0.905
iLJ478	0.955	0.927	0.899	0.896	0.944	0.979	0.879	0.865	0.951	0.95	0.882	0.872
iML1515	0.95	0.946	0.891	0.884	0.983	0.935	0.941	0.942	0.967	0.95	0.916	0.912
iMM1415	0.967	0.961	0.915	0.911	0.967	0.958	0.904	0.898	0.961	0.926	0.902	0.900
iMM904	0.945	0.691	0.802	0.830	0.97	0.948	0.908	0.905	0.962	0.908	0.877	0.876
iND750	0.986	0.984	0.911	0.904	0.968	0.974	0.858	0.839	0.959	0.934	0.873	0.866
iNF517	0.982	0.969	0.912	0.906	0.983	0.969	0.935	0.932	0.982	0.954	0.927	0.925
iNJ661	0.975	0.964	0.914	0.909	0.967	0.938	0.893	0.888	0.964	0.93	0.895	0.889
iNRG857_1313	0.937	0.881	0.863	0.860	0.978	0.931	0.925	0.924	0.963	0.935	0.907	0.904
iPC815	0.934	0.878	0.86	0.857	0.976	0.865	0.899	0.902	0.949	0.908	0.886	0.883
iRC1080	0.942	0.997	0.752	0.671	0.959	0.997	0.806	0.760	0.968	0.972	0.863	0.835
iSB619	0.975	0.948	0.943	0.943	0.982	0.958	0.939	0.938	0.975	0.944	0.931	0.930
iSbBS512_1146	0.93	0.925	0.876	0.869	0.929	0.933	0.898	0.894	0.95	0.935	0.901	0.897
iSBO_1134	0.959	0.869	0.886	0.889	0.974	0.942	0.922	0.921	0.962	0.914	0.906	0.906
iSDY_1059	0.933	0.948	0.893	0.886	0.968	0.971	0.924	0.920	0.931	0.882	0.862	0.865
iSFV_1184	0.982	0.971	0.945	0.944	0.937	0.95	0.907	0.903	0.964	0.946	0.92	0.917
iSFxv_1172	0.953	0.973	0.882	0.870	0.975	0.96	0.932	0.930	0.962	0.953	0.911	0.906
iSF_1195	0.98	0.96	0.929	0.927	0.955	0.927	0.911	0.909	0.953	0.945	0.909	0.905
iSSON_1240	0.949	0.95	0.904	0.899	0.986	0.96	0.944	0.943	0.968	0.952	0.924	0.922
iS_1188	0.98	0.938	0.929	0.928	0.969	0.94	0.907	0.903	0.96	0.926	0.901	0.898
iUMN146_1321	0.967	0.981	0.925	0.921	0.973	0.875	0.909	0.913	0.974	0.929	0.922	0.922

	fold3				fold4				all-fold			
GEMs	AUPRC	Recall	F1 score	Accuracy	AUPRC	Recall	F1 score	Accuracy	AUPRC	Recall	F1 score	Accuracy
iUMNK88_1353	0.983	0.938	0.938	0.938	0.947	0.963	0.886	0.876	0.963	0.951	0.91	0.905
iUTI89_1310	0.981	0.942	0.928	0.927	0.98	0.931	0.929	0.929	0.974	0.945	0.922	0.920
iWFL_1372	0.954	0.938	0.905	0.901	0.975	0.96	0.925	0.922	0.969	0.95	0.922	0.920
iY75_1357	0.952	0.956	0.899	0.893	0.946	0.94	0.893	0.888	0.966	0.946	0.916	0.913
iYL1228	0.96	0.951	0.907	0.902	0.965	0.945	0.906	0.902	0.95	0.879	0.866	0.870
iYO844	0.975	0.979	0.908	0.901	0.931	0.953	0.88	0.870	0.954	0.919	0.878	0.874
iYS1720	0.929	0.905	0.865	0.859	0.976	0.925	0.913	0.912	0.967	0.931	0.914	0.912
iYS854	0.956	0.955	0.854	0.837	0.958	0.931	0.889	0.884	0.958	0.934	0.89	0.883
iZ_1308	0.934	0.948	0.875	0.865	0.983	0.923	0.928	0.928	0.961	0.932	0.9	0.895
RECON1	0.988	0.945	0.941	0.940	0.985	0.932	0.933	0.933	0.986	0.945	0.94	0.940
Recon3D	0.972	0.979	0.921	0.916	0.982	0.954	0.939	0.938	0.976	0.961	0.925	0.922
STM_v1_0	0.968	0.952	0.934	0.932	0.982	0.963	0.938	0.936	0.966	0.922	0.912	0.911
iCN718	0.955	0.947	0.898	0.912	0.945	0.917	0.928	0.911	0.956	0.932	0.913	0.912
iAB_RBC_283	0.921	0.859	0.846	0.844	0.954	0.875	0.875	0.875	0.946	0.866	0.863	0.862
iSynCJ816	0.925	0.943	0.799	0.782	0.922	0.942	0.853	0.860	0.927	0.936	0.843	0.829
e_coli_core	0.919	0.75	0.8	0.813	0.948	0.875	0.875	0.875	0.894	0.875	0.858	0.856

Supplementary Table S4: Details of organisms on 108 BiGG GEMs.

GEMs	Organisms	Categories
e_coli_core	*Escherichia coli* str. K-12 substr. MG1655	prokaryote
iAB_RBC_283	*Homo sapiens*	eukaryote
iIS312	*Trypanosoma cruzi* Dm28c	eukaryote
iIS312_Amastigote	*Trypanosoma cruzi* Dm28c	eukaryote
iIS312_Epimastigote	*Trypanosoma cruzi* Dm28c	eukaryote
iIS312_Trypomastigote	*Trypanosoma cruzi* Dm28c	eukaryote
iIT341	*Helicobacter pylori* 26695	prokaryote
iLJ478	*Thermotoga maritima* MSB8	prokaryote
iAF692	*Methanosarcina barkeri* str. Fusaro	prokaryote
iSB619	*Staphylococcus aureus* subsp. aureus N315	prokaryote
iNF517	*Lactococcus lactis* subsp. cremoris MG1363	prokaryote
iHN637	*Clostridium ljungdahlii* DSM 13528	prokaryote
iJB785	*Synechococcus elongatus* PCC 7942	prokaryote
iJN678	*Synechocystis* sp. PCC 6803	prokaryote
iAT_PLT_636	*Homo sapiens*	eukaryote
iCN718	*Acinetobacter baumannii* AYE	prokaryote
iNJ661	*Mycobacterium tuberculosis* H37Rv	prokaryote
iSynCJ816	*Synechocystis* sp. PCC 6803	prokaryote
iJN746	*Pseudomonas putida* KT2440	prokaryote
iAM_Pb448	*Plasmodium berghei*	eukaryote
iAM_Pc455	*Plasmodium cynomolgi* strain B	eukaryote
iJR904	*Escherichia coli* str. K-12 substr. MG1655	prokaryote
iAM_Pv461	*Plasmodium vivax* Sal-1	eukaryote
iAM_Pk459	*Plasmodium knowlesi* strain H	eukaryote
iAM_Pf480	*Plasmodium falciparum* 3D7	eukaryote
iEK1008	*Mycobacterium tuberculosis* H37Rv	prokaryote
iCN900	*Clostridioides difficile* 630	prokaryote
iYO844	*Bacillus subtilis* subsp. subtilis str. 168	prokaryote

GEMs	Organisms	Categories
iND750	*Saccharomyces cerevisiae* S288C	eukaryotes
iAF987	*Geobacter metallireducens* GS-15	prokaryotes
iYS854	*Staphylococcus aureus* subsp. aureus USA300_TCH1516	prokaryotes
iMM904	*Saccharomyces cerevisiae* S288C	eukaryotes
iPC815	*Yersinia pestis* CO92	prokaryotes
iRC1080	*Chlamydomonas reinhardtii*	eukaryotes
iYL1228	*Klebsiella pneumoniae* subsp. pneumoniae MGH 78578	prokaryotes
iAF1260	*Escherichia coli* str. K-12 substr. MG1655	prokaryotes
iAF1260b	*Escherichia coli* str. K-12 substr. MG1655	prokaryotes
iSDY_1059	*Shigella dysenteriae* Sd197	prokaryotes
STM_v1_0	*Salmonella enterica* subsp. enterica serovar Typhimurium str. LT2	prokaryotes
iJO1366	*Escherichia coli* str. K-12 substr. MG1655	prokaryotes
iSbBS512_1146	*Shigella boydii* CDC 3083-94	prokaryotes
iSBO_1134	*Shigella boydii* Sb227	prokaryotes
iS_1188	*Shigella flexneri* 2a str. 2457T	prokaryotes
iSFV_1184	*Shigella flexneri* 5 str. 8401	prokaryotes
iSF_1195	*Shigella flexneri* 2a str. 301	prokaryotes
iSFxv_1172	*Shigella flexneri* 2002017	prokaryotes
iSSON_1240	*Shigella sonnei* Ss046	prokaryotes
iECH74115_1262	*Escherichia coli* O157:H7 str. EC4115	prokaryotes
iE2348C_1286	*Escherichia coli* O127:H6 str. E2348/69	prokaryotes
iG2583_1286	*Escherichia coli* O55:H7 str. CB9615	prokaryotes
iECED1_1282	*Escherichia coli* ED1a	prokaryotes
iECSP_1301	*Escherichia coli* O157:H7 str. TW14359	prokaryotes
iML1515	*Escherichia coli* str. K-12 substr. MG1655	prokaryotes
iEC042_1314	*Escherichia coli* 042	prokaryotes
iECNA114_1301	*Escherichia coli* NA114	prokaryotes
iECs_1301	*Escherichia coli* O157:H7 str. Sakai	prokaryotes
iECIAI39_1322	*Escherichia coli* IAI39	prokaryotes

GEMs	Organisms	Categories
iZ_1308	*Escherichia coli* O157:H7 str. EDL933	prokaryotes
iUTI89_1310	*Escherichia coli* UTI89	prokaryotes
ic_1306	*Escherichia coli* CFT073	prokaryotes
iEC1344_C	*Escherichia coli* C	prokaryotes
iLF82_1304	*Escherichia coli* LF82	prokaryotes
iECOK1_1307	*Escherichia coli* IHE3034	prokaryotes
iECS88_1305	*Escherichia coli* S88	prokaryotes
iECABU_c1320	*Escherichia coli* ABU 83972	prokaryotes
iAPECO1_1312	*Escherichia coli* APEC O1	prokaryotes
iNRG857_1313	*Escherichia coli* O83:H1 str. NRG 857C	prokaryotes
iUMN146_1321	*Escherichia coli* UM146	prokaryotes
iECP_1309	*Escherichia coli* 536	prokaryotes
iEC1356_Bl21DE3	*Escherichia coli* BL21(DE3)	prokaryotes
iECUMN_1333	*Escherichia coli* UMN026	prokaryotes
iB21_1397	*Escherichia coli* BL21(DE3)	prokaryotes
iBWG_1329	*Escherichia coli* BW2952	prokaryotes
iECD_1391	*Escherichia coli* BL21(DE3)	prokaryotes
iECDH10B_1368	*Escherichia coli* str. K-12 substr. DH10B	prokaryotes
iECSF_1327	*Escherichia coli* SE15	prokaryotes
iEcSMS35_1347	*Escherichia coli* SMS-3-5	prokaryotes
iECB_1328	*Escherichia coli* B str. REL606	prokaryotes
iECBD_1354	*Escherichia coli* 'BL21-Gold(DE3)pLysS AG'	prokaryotes
iEcDH1_1363	*Escherichia coli* DH1	prokaryotes
iEcHS_1320	*Escherichia coli* HS	prokaryotes
iECDH1ME8569_1439	*Escherichia coli* DH1	prokaryotes
iEC1349_Crooks	*Escherichia coli* ATCC 8739	prokaryotes
iEC55989_1330	*Escherichia coli* 55989	prokaryotes
iETEC_1333	*Escherichia coli* ETEC H10407	prokaryotes
iEC1372_W3110	*Escherichia coli* str. K-12 substr. W3110	prokaryotes
iECO103_1326	*Escherichia coli* O103:H2 str. 12009	prokaryotes

GEMs	Organisms	Categories
iY75_1357	*Escherichia coli* str. K-12 substr. W3110	prokaryotes
iECO111_1330	*Escherichia coli* O111:H- str. 11128	prokaryotes
iEcE24377_1341	*Escherichia coli* O139:H28 str. E24377A	prokaryotes
iEC1364_W	*Escherichia coli* W	prokaryotes
iECIAI1_1343	*Escherichia coli* IAI1	prokaryotes
iEcolC_1368	*Escherichia coli* ATCC 8739	prokaryotes
iECSE_1348	*Escherichia coli* SE11	prokaryotes
iUMNK88_1353	*Escherichia coli* UMNK88	prokaryotes
iEKO11_1354	*Escherichia coli* KO11FL	prokaryotes
iEC1368_DH5a	*Escherichia coli* DH5[alpha]	prokaryotes
iECO26_1355	*Escherichia coli* O26:H11 str. 11368	prokaryotes
iECW_1372	*Escherichia coli* W	prokaryotes
iWFL_1372	*Escherichia coli* W	prokaryotes
iJN1463	*Pseudomonas putida* KT2440	prokaryotes
iYS1720	*Salmonella pan-reactome*	prokaryotes
iMM1415	*Mus musculus*	eukaryotes
RECON1	*Homo sapiens*	eukaryotes
iCHOv1_DG44	*Cricetulus griseus*	eukaryotes
iLB1027_lipid	*Phaeodactylum tricornutum* CCAP 1055/1	eukaryotes
iCHOv1	*Cricetulus griseus*	eukaryotes
Recon3D	*Homo sapiens*	eukaryotes

Supplementary Table S5: Detailed reaction recovery performances of HGLMA on 108 BiGG GEMs, where candidate reactions include the positive testing samples with the same number of BiGG reactions.

GEMs	Top 25	Top 50	Top 100	Top *N*
e_coli_core	0.640	0.683	0.663	0.640
iAB_RBC_283	0.677	0.681	0.586	0.629
iAF1260	0.680	0.612	0.684	0.695
iAF1260b	0.645	0.621	0.617	0.641
iAF692	0.756	0.602	0.648	0.585
iAF987	0.676	0.734	0.607	0.651
iAM_Pb448	0.685	0.660	0.600	0.616
iAM_Pc455	0.681	0.643	0.629	0.577
iAM_Pf480	0.675	0.621	0.614	0.650
iAM_Pk459	0.640	0.716	0.572	0.612
iAM_Pv461	0.635	0.647	0.607	0.669
iAPECO1_1312	0.675	0.685	0.621	0.587
iAT_PLT_636	0.638	0.665	0.613	0.640
iB21_1397	0.708	0.637	0.630	0.637
iBWG_1329	0.709	0.628	0.623	0.661
ic_1306	0.714	0.694	0.668	0.639
iCHOv1	0.752	0.664	0.667	0.667
iCHOv1_DG44	0.719	0.632	0.664	0.630
iCN718	0.673	0.676	0.628	0.672
iCN900	0.670	0.679	0.690	0.640
iE2348C_1286	0.725	0.662	0.667	0.644
iEC042_1314	0.676	0.659	0.638	0.583
iEC1344_C	0.718	0.634	0.584	0.612
iEC1349_Crooks	0.710	0.682	0.641	0.670
iEC1356_Bl21DE3	0.675	0.676	0.633	0.595
iEC1364_W	0.678	0.724	0.636	0.603
iEC1368_DH5a	0.711	0.657	0.639	0.654
iEC1372_W3110	0.719	0.669	0.671	0.659
iEC55989_1330	0.714	0.718	0.591	0.655
iECABU_c1320	0.796	0.660	0.615	0.635
iECB_1328	0.714	0.706	0.634	0.657
iECBD_1354	0.687	0.675	0.584	0.653
iECD_1391	0.677	0.643	0.696	0.673
iEcDH1_1363	0.632	0.648	0.639	0.672
iECDH10B_1368	0.681	0.733	0.591	0.713
iECDH1ME8569_1439	0.671	0.702	0.711	0.652
iEcE24377_1341	0.678	0.703	0.627	0.667
iECED1_1282	0.667	0.725	0.692	0.615
iECH74115_1262	0.751	0.655	0.599	0.663
iEcHS_1320	0.678	0.695	0.644	0.662
iECIAI1_1343	0.681	0.624	0.643	0.605
iECIAI39_1322	0.718	0.683	0.623	0.641
iECNA114_1301	0.677	0.683	0.679	0.637

GEMs	Top 25	Top 50	Top 100	Top N
iECO103_1326	0.716	0.703	0.581	0.698
iECO111_1330	0.670	0.638	0.652	0.676
iECO26_1355	0.674	0.683	0.627	0.599
iECOK1_1307	0.675	0.663	0.661	0.661
iEcolC_1368	0.723	0.698	0.602	0.585
iECP_1309	0.639	0.651	0.646	0.667
iECs_1301	0.708	0.664	0.611	0.629
iECS88_1305	0.719	0.595	0.645	0.608
iECSE_1348	0.755	0.665	0.620	0.667
iECSF_1327	0.674	0.717	0.625	0.634
iEcSMS35_1347	0.720	0.705	0.637	0.605
iECSP_1301	0.722	0.627	0.644	0.685
iECUMN_1333	0.722	0.677	0.560	0.641
iECW_1372	0.673	0.686	0.633	0.630
iEK1008	0.716	0.704	0.637	0.651
iEKO11_1354	0.759	0.686	0.572	0.632
iETEC_1333	0.722	0.691	0.669	0.595
iG2583_1286	0.715	0.700	0.638	0.627
iHN637	0.676	0.618	0.634	0.612
iIS312	0.672	0.697	0.647	0.633
iIS312_Amastigote	0.711	0.657	0.665	0.678
iIS312_Epimastigote	0.641	0.701	0.597	0.627
iIS312_Trypomastigote	0.644	0.623	0.620	0.599
iIT341	0.717	0.643	0.645	0.588
iJB785	0.722	0.686	0.631	0.602
iJN1463	0.679	0.702	0.637	0.639
iJN678	0.754	0.709	0.627	0.709
iJN746	0.719	0.680	0.546	0.627
iJO1366	0.685	0.715	0.600	0.663
iJR904	0.677	0.645	0.578	0.633
iLB1027_lipid	0.685	0.737	0.604	0.731
iLF82_1304	0.624	0.676	0.676	0.636
iLJ478	0.641	0.664	0.634	0.680
iML1515	0.718	0.640	0.674	0.664
iMM1415	0.716	0.725	0.641	0.613
iMM904	0.678	0.724	0.659	0.680
iND750	0.707	0.701	0.633	0.683
iNF517	0.756	0.657	0.621	0.592
iNJ661	0.719	0.634	0.668	0.594
iNRG857_1313	0.765	0.739	0.591	0.629
iPC815	0.715	0.686	0.590	0.611
iRC1080	0.673	0.663	0.583	0.670
iS_1188	0.715	0.675	0.634	0.622
iSB619	0.682	0.702	0.641	0.686
iSbBS512_1146	0.679	0.683	0.620	0.669
iSBO_1134	0.715	0.677	0.637	0.651

GEMs	Top 25	Top 50	Top 100	Top N
iSDY_1059	0.680	0.642	0.647	0.651
iSF_1195	0.678	0.722	0.643	0.620
iSFV_1184	0.682	0.656	0.694	0.630
iSFxv_1172	0.754	0.703	0.660	0.661
iSSON_1240	0.716	0.684	0.636	0.657
iSynCJ816	0.715	0.667	0.616	0.636
iUMN146_1321	0.710	0.647	0.602	0.650
iUMNK88_1353	0.719	0.655	0.631	0.686
iUTI89_1310	0.679	0.677	0.631	0.637
iWFL_1372	0.757	0.644	0.623	0.655
iY75_1357	0.756	0.684	0.617	0.654
iYL1228	0.710	0.704	0.640	0.660
iYO844	0.715	0.701	0.625	0.626
iYS1720	0.676	0.704	0.618	0.597
iYS854	0.673	0.647	0.606	0.688
iZ_1308	0.636	0.666	0.657	0.649
RECON1	0.719	0.745	0.610	0.609
Recon3D	0.687	0.680	0.633	0.656
STM_v1_0	0.638	0.665	0.657	0.644

Supplementary Table S6: Detailed reaction recovery performances of HGLMA on 108 BiGG GEMs, where candidate reactions include the positive testing samples with the same number of BiGG reactions.

GEMs	Top 25	Top 50	Top 100	Top N
e_coli_core	0.170	0.234	0.143	0.118
iAB_RBC_283	0.166	0.153	0.226	0.117
iAF1260	0.170	0.203	0.145	0.129
iAF1260b	0.255	0.193	0.157	0.127
iAF692	0.166	0.153	0.259	0.141
iAF987	0.166	0.265	0.138	0.126
iAM_Pb448	0.175	0.152	0.210	0.123
iAM_Pc455	0.211	0.154	0.139	0.119
iAM_Pf480	0.165	0.153	0.205	0.123
iAM_Pk459	0.210	0.147	0.162	0.176
iAM_Pv461	0.205	0.179	0.147	0.199
iAPECO1_1312	0.205	0.197	0.152	0.146
iAT_PLT_636	0.248	0.157	0.223	0.129
iB21_1397	0.157	0.188	0.250	0.161
iBWG_1329	0.238	0.200	0.183	0.125
ic_1306	0.244	0.206	0.248	0.120
iCHOv1	0.242	0.256	0.247	0.213
iCHOv1_DG44	0.249	0.264	0.224	0.124
iCN718	0.242	0.248	0.188	0.124
iCN900	0.240	0.170	0.250	0.213
iE2348C_1286	0.254	0.233	0.137	0.121
iEC042_1314	0.246	0.190	0.148	0.124
iEC1344_C	0.207	0.145	0.244	0.210
iEC1349_Crooks	0.240	0.253	0.251	0.206
iEC1356_Bl21DE3	0.164	0.188	0.223	0.197
iEC1364_W	0.208	0.255	0.146	0.181
iEC1368_DH5a	0.201	0.228	0.249	0.210
iEC1372_W3110	0.169	0.261	0.251	0.169
iEC55989_1330	0.204	0.189	0.241	0.221
iECABU_c1320	0.246	0.191	0.155	0.213
iECB_1328	0.204	0.177	0.245	0.217
iECBD_1354	0.216	0.147	0.244	0.118
iECD_1391	0.247	0.254	0.176	0.123
iEcDH1_1363	0.162	0.239	0.219	0.187
iECDH10B_1368	0.251	0.225	0.141	0.199
iECDH1ME8569_1439	0.161	0.254	0.252	0.138
iEcE24377_1341	0.168	0.254	0.258	0.220
iECED1_1282	0.157	0.257	0.152	0.221
iECH74115_1262	0.160	0.247	0.229	0.179
iEcHS_1320	0.208	0.226	0.225	0.219
iECIAI1_1343	0.211	0.156	0.153	0.220
iECIAI39_1322	0.208	0.254	0.143	0.187
iECNA114_1301	0.246	0.154	0.249	0.177

GEMs	Top 25	Top 50	Top 100	Top N
iECO103_1326	0.246	0.195	0.211	0.147
iECO111_1330	0.200	0.250	0.202	0.124
iECO26_1355	0.203	0.174	0.247	0.173
iECOK1_1307	0.245	0.194	0.171	0.206
iEcolC_1368	0.172	0.249	0.182	0.163
iECP_1309	0.249	0.162	0.246	0.161
iECs_1301	0.158	0.235	0.241	0.130
iECS88_1305	0.209	0.187	0.255	0.134
iECSE_1348	0.245	0.196	0.220	0.213
iECSF_1327	0.164	0.168	0.245	0.205
iEcSMS35_1347	0.250	0.237	0.148	0.195
iECSP_1301	0.212	0.179	0.254	0.180
iECUMN_1333	0.252	0.149	0.170	0.213
iECW_1372	0.203	0.238	0.183	0.186
iEK1008	0.205	0.155	0.177	0.214
iEKO11_1354	0.249	0.237	0.242	0.112
iETEC_1333	0.212	0.262	0.249	0.120
iG2583_1286	0.165	0.192	0.238	0.213
iHN637	0.246	0.149	0.214	0.124
iIS312	0.201	0.248	0.147	0.125
iIS312_Amastigote	0.161	0.189	0.145	0.164
iIS312_Epimastigote	0.171	0.213	0.228	0.144
iIS312_Trypomastigote	0.174	0.195	0.150	0.120
iIT341	0.247	0.154	0.155	0.162
iJB785	0.212	0.257	0.191	0.146
iJN1463	0.249	0.193	0.158	0.116
iJN678	0.204	0.220	0.197	0.206
iJN746	0.209	0.152	0.146	0.136
iJO1366	0.175	0.247	0.250	0.215
iJR904	0.247	0.176	0.218	0.114
iLB1027_lipid	0.175	0.169	0.144	0.150
iLF82_1304	0.154	0.168	0.226	0.216
iLJ478	0.251	0.255	0.144	0.223
iML1515	0.247	0.192	0.224	0.193
iMM1415	0.166	0.236	0.151	0.214
iMM904	0.167	0.255	0.149	0.165
iND750	0.157	0.253	0.163	0.192
iNF517	0.206	0.249	0.201	0.215
iNJ661	0.249	0.165	0.148	0.220
iNRG857_1313	0.214	0.191	0.151	0.212
iPC815	0.164	0.157	0.250	0.175
iRC1080	0.243	0.254	0.243	0.218
iS_1188	0.205	0.147	0.204	0.212
iSB619	0.212	0.254	0.151	0.207
iSbBS512_1146	0.209	0.195	0.160	0.211
iSBO_1134	0.204	0.169	0.177	0.201

GEMs	Top 25	Top 50	Top 100	Top N
iSDY_1059	0.250	0.174	0.148	0.143
iSF_1195	0.248	0.233	0.153	0.216
iSFV_1184	0.252	0.207	0.154	0.199
iSFxv_1172	0.204	0.215	0.240	0.225
iSSON_1240	0.245	0.156	0.236	0.218
iSynCJ816	0.205	0.198	0.186	0.216
iUMN146_1321	0.240	0.198	0.232	0.123
iUMNK88_1353	0.209	0.226	0.181	0.227
iUTI89_1310	0.168	0.248	0.251	0.140
iWFL_1372	0.247	0.216	0.233	0.169
iY75_1357	0.246	0.215	0.247	0.128
iYL1228	0.160	0.156	0.250	0.126
iYO844	0.165	0.212	0.225	0.172
iYS1720	0.205	0.216	0.239	0.118
iYS854	0.163	0.239	0.146	0.218
iZ_1308	0.206	0.178	0.217	0.208
RECON1	0.169	0.256	0.180	0.208
Recon3D	0.256	0.211	0.243	0.211
STM_v1_0	0.208	0.177	0.247	0.113

Supplementary Table S7: Details of 1D and 2D weight scores of trained HGLMA on 108 BiGG GEMs.

GEMs	Normalized 1D weight scores	Normalized 2D weight scores
RECON1	0.617898978	0.382101022
Recon3D	0.628983905	0.371016095
STM_v1_0	0.624120438	0.375879562
e_coli_core	0.547338581	0.452661419
iAB_RBC_283	0.57647677	0.42352323
iAF1260	0.608546315	0.391453685
iAF1260b	0.628364026	0.371635974
iAF692	0.560743609	0.439256391
iAF987	0.639546039	0.360453961
iAM_Pb448	0.564543397	0.435456603
iAM_Pc455	0.576483925	0.423516075
iAM_Pf480	0.57390919	0.42609081
iAM_Pk459	0.571588144	0.428411856
iAM_Pv461	0.553728495	0.446271505
iAPECO1_1312	0.636447047	0.363552953
iAT_PLT_636	0.573262401	0.426737599
iB21_1397	0.627508729	0.372491271
iBWG_1329	0.625486217	0.374513783
iCHOv1	0.629463354	0.370536646
iCHOv1_DG44	0.615296181	0.384703819
iCN718	0.62889441	0.37110559
iCN900	0.603575136	0.396424864
iE2348C_1286	0.596842079	0.403157921
iEC042_1314	0.588045249	0.411954751
iEC1344_C	0.610772656	0.389227344
iEC1349_Crooks	0.632606974	0.367393026
iEC1356_Bl21DE3	0.623997453	0.376002547
iEC1364_W	0.634524124	0.365475876
iEC1368_DH5a	0.627775833	0.372224167
iEC1372_W3110	0.605011018	0.394988982
iEC55989_1330	0.630823489	0.369176511
iECABU_c1320	0.637121727	0.362878273
iECBD_1354	0.622244137	0.377755863
iECB_1328	0.639218855	0.360781145
iECDH10B_1368	0.606686216	0.393313784
iECDH1ME8569_1439	0.62253055	0.37746945
iECD_1391	0.63957932	0.36042068
iECED1_1282	0.616095516	0.383904484
iECH74115_1262	0.629068237	0.370931763
iECIAI1_1343	0.60991738	0.39008262
iECIAI39_1322	0.629148067	0.370851933
iECNA114_1301	0.617232679	0.382767321
iECO103_1326	0.622523317	0.377476683
iECO111_1330	0.62900293	0.37099707
iECO26_1355	0.643056601	0.356943399

GEMs	Normalized 1D weight scores	Normalized 2D weight scores
iECOK1_1307	0.631878019	0.368121981
iECP_1309	0.637722574	0.362277426
iECS88_1305	0.606441681	0.393558319
iECSE_1348	0.63691474	0.36308526
iECSF_1327	0.630441829	0.369558171
iECSP_1301	0.635670794	0.364329206
iECUMN_1333	0.617670181	0.382329819
iECW_1372	0.60218778	0.39781222
iECs_1301	0.627483936	0.372516064
iEK1008	0.608695781	0.391304219
iEKO11_1354	0.63087875	0.36912125
iETEC_1333	0.623647076	0.376352924
iEcDH1_1363	0.628946946	0.371053054
iEcE24377_1341	0.623817107	0.376182893
iEcHS_1320	0.63255305	0.36744695
iEcSMS35_1347	0.630199242	0.369800758
iEcolC_1368	0.633028094	0.366971906
iG2583_1286	0.62716953	0.37283047
iHN637	0.580286706	0.419713294
iIS312	0.527726997	0.472273003
iIS312_Amastigote	0.553706541	0.446293459
iIS312_Epimastigote	0.524780437	0.475219563
iIS312_Trypomastigote	0.529728137	0.470271863
iIT341	0.536751651	0.463248349
iJB785	0.584160994	0.415839006
iJN1463	0.646205067	0.353794933
iJN678	0.595337515	0.404662485
iJN746	0.59903641	0.40096359
iJO1366	0.623242127	0.376757873
iJR904	0.600394678	0.399605322
iLB1027_lipid	0.627026211	0.372973789
iLF82_1304	0.627630967	0.372369033
iLJ478	0.554511183	0.445488817
iML1515	0.634651085	0.365348915
iMM1415	0.590578024	0.409421976
iMM904	0.64061068	0.35938932
iND750	0.61712463	0.38287537
iNF517	0.560112876	0.439887124
iNJ661	0.593835322	0.406164678
iNRG857_1313	0.63111261	0.36888739
iPC815	0.59640027	0.40359973
iRC1080	0.610436514	0.389563486
iSB619	0.579953229	0.420046771
iSBO_1134	0.629037724	0.370962276
iSDY_1059	0.601468909	0.398531091
iSFV_1184	0.62186672	0.37813328

GEMs	Normalized 1D weight scores	Normalized 2D weight scores
iSF_1195	0.639249477	0.360750523
iSFxv_1172	0.627347695	0.372652305
iSSON_1240	0.637367463	0.362632537
iS_1188	0.62678544	0.37321456
iSbBS512_1146	0.624674377	0.375325623
iSynCJ816	0.599101371	0.400898629
iUMN146_1321	0.626427434	0.373572566
iUMNK88_1353	0.633268282	0.366731718
iUTI89_1310	0.637154825	0.362845175
iWFL_1372	0.631352329	0.368647671
iY75_1357	0.640293028	0.359706972
iYL1228	0.628535588	0.371464412
iYO844	0.596002759	0.403997241
iYS1720	0.631838411	0.368161589
iYS854	0.613516779	0.386483221
iZ_1308	0.620135565	0.379864435
ic_1306	0.631876256	0.368123744

Supplementary Table S8. Bacterial genomes used in our testing of fermentation products.

NCBI Assembly	Taxonomy
GCF_000005845.2	*Escherichia coli* str. K-12 substr. MG1655
GCF_000008345.1	*Cutibacterium acnes* KPA171202
GCF_000008545.1	*Thermotoga maritima* MSB8
GCF_000008765.1	*Clostridium acetobutylicum* ATCC 824
GCF_000011065.1	*Bacteroides thetaiotaomicron* VPI-5482
GCF_000011985.1	*Lactobacillus acidophilus* NCFM
GCF_000013285.1	*Clostridium perfringens* ATCC 13124
GCF_000020425.1	*Bifidobacterium longum* subsp. infantis ATCC 15697
GCF_000020605.1	*Eubacterium rectale* ATCC 33656
GCF_000022965.1	*Bifidobacterium animalis* subsp. lactis DSM 10140
GCF_000025885.1	*Aminobacterium colombiense* DSM 12261
GCF.000056065.1	*Lactobacillus delbruecki* subsp. bulgaricus ATCC 11842
GCF_000143845.1	*Olsenella uli* DSM 7084
GCF_000144405.1	*Prevotella melaninogenica* ATCC 25845
GCF_000160535.1	*Prevotella bergensis* DSM 17361
GCF_000173975.1	*Anaerobutyricum halli* DSM 3353
GCF_000175255.2	*Zymomonas mobilis* subsp. mobilis ATCC 10988
GCF_000389635.1	*Clostridium pasteurianum* BC1
GCF_000392875.1	*Enterococcus faecalis* ATCC 19433
GCF_000005845.2	*Eubacterium ramulus* ATCC 29099
GCF 001456065.2	*Clostridium butyricum* KNU-L09
GCF 001561955.1	*Anaerotignum propionicum* DSM 1682
GCF 000162015.1	*Faecalibacterium prausnitzii* A2-165
GCF 000203855.3	*Lactobacillus plantarum* WCFS1

Supplementary Table S9: Reported data of fermentation end products by experiments for 24 baterial organisms.

Organism names	Reported fermentation end products
Escherichia coli str. K-12 substr. MG1655	acetic acid, formic acid, ethanol, succinic acid,, DL-lactic acid
Cutibacterium acnes KPA171202	propionic acid, acetic acid, DL-lactic acid
Thermotoga maritima MSB8	acetic acid
Clostridium acetobutylicum ATCC 824	butyric acid, n-butanol, ethanol, acetic acid, acetone
Bacteroides thetaiotaomicron VPI-5482	acetic acid, propionic acid, formic acid
Lactobacillus acidophilus NCFM	acetic acid, DL-lactic acid
Clostridium perfringens ATCC 13124	butyric acid, acetic acid, ethanol, DL-lactic acid, propionic acid, formic acid
Bifidobacterium longum subsp. infantis ATCC 15697	acetic acid, DL-lactic acid
Eubacterium rectale ATCC 33656	butyric acid, formic acid, DL-lactic acid
Bifidobacterium animalis subsp. lactis DSM 10140	acetic acid, DL-lactic acid
Aminobacterium colombiense DSM 12261	propionic acid, acetic acid
Lactobacillus delbruecki subsp. bulgaricus ATCC 11842	DL-lactic acid, acetic acid
Olsenella uli DSM 7084	DL-lactic acid, acetic acid, formic acid
Prevotella melaninogenica ATCC 25845	acetic acid, succinic acid
Prevotella bergensis DSM 17361	acetic acid, succinic acid
Anaerobutyricum halli DSM 3353	butyric acid
Zymomonas mobilis subsp. mobilis ATCC 10988	ethanol
Clostridium pasteurianum BC1	acetic acid, butyric acid
Enterococcus faecalis ATCC 19433	acetic acid, formic acid, DL-lactic acid, ethanol
Eubacterium ramulus ATCC 29099	butyric acid, acetic acid, ethanol
Clostridium butyricum KNU-L09	acetic acid, butyric acid, formic acid
Anaerotignum propionicum DSM 1682	propionic acid, acetic acid
Faecalibacterium prausnitzii A2-165	butyric acid, DL-lactic acid, formic acid
Lactobacillus plantarum WCFS1	acetic acid, DL-lactic acid

Supplementary Table S10: Details of predicted fermentation end products by gap-filled GEMs (HGLMA) for 24 bacterial organisms.

Organism names	Predicted fermentation end products
Escherichia coli str. K-12 substr. MG1655	acetic acid, ethanol, formic acid, DL-lactic acid, succinic acid
Cutibacterium acnes KPA171202	acetic acid, formic acid
Thermotoga maritima MSB8	succinic acid
Clostridium acetobutylicum ATCC 824	acetic acid, DL-lactic acid, propionic acid, succinic acid
Bacteroides thetaiotaomicron VPI-5482	acetic acid, ethanol, formic acid, DL-lactic acid
Lactobacillus acidophilus NCFM	acetic acid, DL-lactic acid
Clostridium perfringens ATCC 13124	ethanol, formic acid, DL-lactic acid, succinic acid
Bifidobacterium longum subsp. infantis ATCC 15697	acetic acid, ethanol, formic acid, DL-lactic acid, succinic acid
Eubacterium rectale ATCC 33656	butyric acid, formic acid, DL-lactic acid, succinic acid
Bifidobacterium animalis subsp. lactis DSM 10140	formic acid, succinic acid
Aminobacterium colombiense DSM 12261	ethanol, formic acid, DL-lactic acid, propionic acid, succinic acid
Lactobacillus delbruecki subsp. bulgaricus ATCC 11842	ethanol, formic acid, DL-lactic acid, succinic acid
Olsenella uli DSM 7084	acetic acid, ethanol, formic acid, DL-lactic acid, succinic acid
Prevotella melaninogenica ATCC 25845	ethanol, succinic acid
Prevotella bergensis DSM 17361	formic acid
Anaerobutyricum halli DSM 3353	formic acid, DL-lactic acid, propionic acid, succinic acid
Zymomonas mobilis subsp. mobilis ATCC 10988	acetic acid, formic acid, DL-lactic acid, succinic acid
Clostridium pasteurianum BC1	formic acid, DL-lactic acid, succinic acid
Enterococcus faecalis ATCC 19433	ethanol, formic acid, DL-lactic acid, succinic acid
Eubacterium ramulus ATCC 29099	acetic acid, ethanol, formic acid, succinic acid
Clostridium butyricum KNU-L09	acetic acid, ethanol, formic acid, DL-lactic acid, succinic acid
Anaerotignum propionicum DSM 1682	butyric acid, ethanol, formic acid, DL-lactic acid, propionic acid, succinic acid
Faecalibacterium prausnitzii A2-165	ethanol, formic acid, succinic acid
Lactobacillus plantarum WCFS1	acetic acid, formic acid, DL-lactic acid, succinic acid

Supplementary Table S11: Performance metric values of phenotypic prediction results by draft GEMs and gap-filled GEMs (HGLMA) for 24 bacterial organisms.

	GEM (Draft)				GEM (HGLMA)			
Organism names	AUPRC	Recall	F1 Score	Precision	AUPRC	Recall	F1 Score	Precision
Aminobacterium colombiense DSM 12261	0.2222	0.0000	0.0000	0.0000	0.2111	0.5000	0.2857	0.2000
Anaerobutyricum halli DSM 3353	0.1111	0.0000	0.0000	0.0000	0.1111	0.0000	0.0000	0.0000
Anaerotignum propionicum DSM 1682	0.2222	0.0000	0.0000	0.0000	0.1944	0.5000	0.2500	0.1667
Bacteroides thetaiotaomicron VPI-5482	0.2222	0.0000	0.0000	0.0000	0.4444	0.6667	0.5714	0.5000
Bifidobacterium animalis subsp. lactis DSM 10140	0.2222	0.0000	0.0000	0.0000	0.2222	0.0000	0.0000	0.0000
Bifidobacterium longum subsp. infantis ATCC 15697	0.6667	0.8000	0.8000	0.8000	0.4000	1.0000	0.5714	0.4000
Clostridium acetobutylicum ATCC 824	0.5556	0.0000	0.0000	0.0000	0.4944	0.2000	0.2222	0.2500
Clostridium butyricum KNU-L09	0.4444	0.6667	0.5714	0.5000	0.3778	0.6667	0.5000	0.4000
Clostridium pasteurianum BC1	0.2222	0.0000	0.0000	0.0000	0.2222	0.0000	0.0000	0.0000
Clostridium perfringens ATCC 13124	0.6389	0.1667	0.2500	0.5000	0.7083	0.5000	0.6000	0.7500
Cutibacterium acnes KPA171202	0.4444	0.6667	0.5714	0.5000	0.3889	0.3333	0.4000	0.5000
Enterococcus faecalis ATCC 19433	0.5556	0.5000	0.3333	0.2500	0.6736	0.7500	0.7500	0.7500
Escherichia coli str. K-12 substr. MG1655	1.0000	1.0000	1.0000	1.0000	1.0000	1.0000	1.0000	1.0000
Eubacterium ramulus ATCC 29099	0.3333	0.0000	0.0000	0.0000	0.4444	0.6667	0.5714	0.5000
Eubacterium rectale ATCC 33656	0.3333	0.0000	0.0000	0.0000	0.7500	1.0000	0.8571	0.7500
Faecalibacterium prausnitzii A2-165	0.3333	0.0000	0.0000	0.0000	0.3333	0.3333	0.3333	0.3333
Lactobacillus acidophilus NCFM	0.3611	0.5000	0.5000	0.5000	1.0000	1.0000	1.0000	1.0000
Lactobacillus delbruecki subsp. bulgaricus ATCC 11842	0.2361	0.5000	0.3333	0.2500	0.2361	0.5000	0.3333	0.2500
Lactobacillus plantarum WCFS1	0.3611	0.5000	0.5000	0.5000	0.5000	1.0000	0.6667	0.5000
Olsenella uli DSM 7084	0.3889	0.3333	0.4000	0.5000	0.6000	1.0000	0.7500	0.6000
Prevotella bergensis DSM 17361	0.2222	0.0000	0.0000	0.0000	0.2222	0.0000	0.0000	0.0000
Prevotella melaninogenica ATCC 25845	0.2222	0.0000	0.0000	0.0000	0.3611	0.5000	0.5000	0.5000
Thermotoga maritima MSB8	0.1111	0.0000	0.0000	0.0000	0.1111	0.0000	0.0000	0.0000
Zymomonas mobilis subsp. mobilis ATCC 10988	0.1111	0.0000	0.0000	0.0000	0.1111	0.0000	0.0000	0.0000

	GEM (Random)				GEM (CHESHIRE)			
Organism names	AUPRC	Recall	F1 Score	Precision	AUPRC	Recall	F1 Score	Precision
Aminobacterium colombiense DSM 12261	0.2222	0.0000	0.0000	0.0000	0.2222	0.0000	0.0000	0.0000
Anaerobutyricum halli DSM 3353	0.1111	0.0000	0.0000	0.0000	0.1111	0.0000	0.0000	0.0000
Anaerotignum propionicum DSM 1682	0.2222	0.0000	0.0000	0.0000	0.2222	0.0000	0.0000	0.0000
Bacteroides thetaiotaomicron VPI-5482	0.2222	0.0000	0.0000	0.0000	0.3333	0.0000	0.0000	0.0000
Bifidobacterium animalis subsp. lactis DSM 10140	0.2222	0.0000	0.0000	0.0000	0.2222	0.0000	0.0000	0.0000
Bifidobacterium longum subsp. infantis ATCC 15697	0.6667	0.8000	0.8000	0.8000	0.6667	1.0000	0.8000	0.6667
Clostridium acetobutylicum ATCC 824	0.5556	0.0000	0.0000	0.0000	0.5556	0.0000	0.0000	0.0000
Clostridium butyricum KNU-L09	0.4444	0.6667	0.5714	0.5000	0.4444	0.6667	0.5714	0.5000
Clostridium pasteurianum BC1	0.2222	0.0000	0.0000	0.0000	0.2222	0.0000	0.0000	0.0000
Clostridium perfringens ATCC 13124	0.6389	0.1667	0.2500	0.5000	0.6389	0.1667	0.2500	0.5000
Cutibacterium acnes KPA171202	0.4444	0.6667	0.5714	0.5000	0.4444	0.6667	0.5714	0.5000
Enterococcus faecalis ATCC 19433	0.5556	0.5000	0.3333	0.2500	0.5556	0.5000	0.5714	0.6667
Escherichia coli str. K-12 substr. MG1655	1.0000	1.0000	1.0000	1.0000	1.0000	1.0000	1.0000	1.0000
Eubacterium ramulus ATCC 29099	0.3333	0.0000	0.0000	0.0000	0.3333	0.0000	0.0000	0.0000
Eubacterium rectale ATCC 33656	0.3333	0.0000	0.0000	0.0000	0.3333	0.0000	0.0000	0.0000
Faecalibacterium prausnitzii A2-165	0.3333	0.0000	0.0000	0.0000	0.3333	0.0000	0.0000	0.0000
Lactobacillus acidophilus NCFM	0.3611	0.5000	0.5000	0.5000	0.3611	0.5000	0.5000	0.5000
Lactobacillus delbruecki subsp. bulgaricus ATCC 11842	0.2361	0.5000	0.3333	0.2500	0.2361	0.5000	0.3333	0.2500
Lactobacillus plantarum WCFS1	0.3611	0.5000	0.5000	0.5000	0.3611	0.5000	0.5000	0.5000
Olsenella uli DSM 7084	0.3889	0.3333	0.4000	0.5000	0.3889	0.3333	0.4000	0.5000
Prevotella bergensis DSM 17361	0.2222	0.0000	0.0000	0.0000	0.2222	0.0000	0.0000	0.0000
Prevotella melaninogenica ATCC 25845	0.2222	0.0000	0.0000	0.0000	0.2222	0.0000	0.0000	0.0000
Thermotoga maritima MSB8	0.1111	0.0000	0.0000	0.0000	0.1111	0.0000	0.0000	0.0000
Zymomonas mobilis subsp. mobilis ATCC 10988	0.1111	0.0000	0.0000	0.0000	0.1111	0.0000	0.0000	0.0000

	GEM (CLOSEgaps)				GEM (MuSHIN)			
Organism names	AUPRC	Recall	F1 Score	Precision	AUPRC	Recall	F1 Score	Precision
Aminobacterium colombiense DSM 12261	0.2222	0.0000	0.0000	0.0000	0.2222	0.0000	0.0000	0.0000
Anaerobutyricum halli DSM 3353	0.1111	0.0000	0.0000	0.0000	0.1111	0.0000	0.0000	0.0000
Anaerotignum propionicum DSM 1682	0.2222	0.0000	0.0000	0.0000	0.2111	0.5000	0.2857	0.2000
Bacteroides thetaiotaomicron VPI-5482	0.3333	0.0000	0.0000	0.0000	0.4444	0.6667	0.5714	0.5000
Bifidobacterium animalis subsp. lactis DSM 10140	0.2222	0.0000	0.0000	0.0000	0.2222	0.0000	0.0000	0.0000
Bifidobacterium longum subsp. infantis ATCC 15697	0.6667	1.0000	0.8000	0.6667	0.5000	1.0000	0.6667	0.5000
Clostridium acetobutylicum ATCC 824	0.5556	0.0000	0.0000	0.0000	0.5556	0.0000	0.0000	0.0000
Clostridium butyricum KNU-L09	0.4444	0.6667	0.5714	0.5000	0.3778	0.6667	0.5000	0.4000
Clostridium pasteurianum BC1	0.2222	0.0000	0.0000	0.0000	0.2222	0.0000	0.0000	0.0000
Clostridium perfringens ATCC 13124	0.7083	0.5000	0.6000	0.7500	0.7083	0.5000	0.6000	0.7500
Cutibacterium acnes KPA171202	0.4444	0.6667	0.5714	0.5000	0.4444	0.6667	0.5714	0.5000
Enterococcus faecalis ATCC 19433	0.5556	0.5000	0.5714	0.6667	0.6736	0.7500	0.7500	0.7500
Escherichia coli str. K-12 substr. MG1655	1.0000	1.0000	1.0000	1.0000	0.8333	1.0000	0.9091	0.8333
Eubacterium ramulus ATCC 29099	0.3889	0.3333	0.4000	0.5000	0.3778	0.6667	0.5000	0.4000
Eubacterium rectale ATCC 33656	0.3889	0.3333	0.4000	0.5000	0.5556	0.6667	0.6667	0.6667
Faecalibacterium prausnitzii A2-165	0.3333	0.3333	0.3333	0.3333	0.3333	0.3333	0.3333	0.3333
Lactobacillus acidophilus NCFM	0.2778	0.5000	0.4000	0.3333	0.6667	1.0000	0.8000	0.6667
Lactobacillus delbruecki subsp. bulgaricus ATCC 11842	0.2778	0.5000	0.4000	0.3333	0.2361	0.5000	0.3333	0.2500
Lactobacillus plantarum WCFS1	0.3611	0.5000	0.5000	0.5000	0.5000	1.0000	0.6667	0.5000
Olsenella uli DSM 7084	0.3333	0.3333	0.3333	0.3333	0.6000	1.0000	0.7500	0.6000
Prevotella bergensis DSM 17361	0.2222	0.0000	0.0000	0.0000	0.2222	0.0000	0.0000	0.0000
Prevotella melaninogenica ATCC 25845	0.2222	0.0000	0.0000	0.0000	0.6111	0.5000	0.6667	1.0000
Thermotoga maritima MSB8	0.1111	0.0000	0.0000	0.0000	0.1111	0.0000	0.0000	0.0000
Zymomonas mobilis subsp. mobilis ATCC 10988	0.1111	0.0000	0.0000	0.0000	0.1111	0.0000	0.0000	0.0000

Supplementary Table S12. Details of the training time and inference time of HGLMA for gap-fillings of 24 draft GEMs in metabolic phenotype prediction, by using Python 3.8 and PyTorch 2.1.0 with CUDA 12.1 and an NVIDIA RTX4090 graphics card.

Taxonomy	Training time (h)	Inference time (s)
Escherichia coli str. K-12 substr. MG1655	2.02	49.1
Cutibacterium acnes KPA171202	1.11	49.2
Thermotoga maritima MSB8	0.52	49.4
Clostridium acetobutylicum ATCC 824	1.01	49.2
Bacteroides thetaiotaomicron VPI-5482	1.91	49.1
Lactobacillus acidophilus NCFM	0.76	49.2
Clostridium perfringens ATCC 13124	0.73	49.2
Bifidobacterium longum subsp. infantis ATCC 15697	1.16	49.2
Eubacterium rectale ATCC 33656	0.90	49.3
Bifidobacterium animalis subsp. lactis DSM 10140	0.64	49.3
Aminobacterium colombiense DSM 12261	0.69	49.3
Lactobacillus delbruecki subsp. bulgaricus ATCC 11842	0.72	49.3
Olsenella uli DSM 7084	0.67	49.3
Prevotella melaninogenica ATCC 25845	0.93	49.2
Prevotella bergensis DSM 17361	0.96	49.2
Anaerobutyricum halli DSM 3353	0.88	49.2
Zymomonas mobilis subsp. mobilis ATCC 10988	0.95	49.1
Clostridium pasteurianum BC1	0.57	49.3
Enterococcus faecalis ATCC 19433	0.98	49.2
Eubacterium ramulus ATCC 29099	0.84	49.2
Clostridium butyricum KNU-L09	0.79	49.2
Anaerotignum propionicum DSM 1682	0.64	49.3
Faecalibacterium prausnitzii A2-165	0.85	49.2
Lactobacillus plantarum WCFS1	1.46	49.1
